# Application of Rational Design and Molecular Metadynamics for the Estimation of Changes in Trans-Splicing Efficiency during the Mutagenesis of Ssp DnaE Intein

**DOI:** 10.1021/acsbiomedchemau.5c00091

**Published:** 2025-08-08

**Authors:** Matvei O. Sabantsev, Andrew N. Brovin, Maxim A. Gureev, Yuri B. Porozov, Sergey A. Chuvpilo, Alexander V. Karabelsky

**Affiliations:** † Gene Therapy Department, Research Center for Translational Medicine, 701584 Sirius University of Science and Technology, Sirius Federal Territory, Krasnodar Region 354349, Russia; ‡ Laboratory of Bio- and Chemoinformatics, 328555 HSE University, Soyuza Pechatnikov Str.16, Saint-Petersburg 190121, Russia

**Keywords:** inteins, protein trans-splicing, rational mutagenesis, protein−protein docking, molecular metadynamics simulation, gene therapy

## Abstract

Currently, inteins are some of the most popular multifunctional tools in the fields of molecular biology and biotechnology. In this study, we used the surface analysis method to identify the sites of intermolecular interactions between the N and C-parts of the Ssp DnaE intein. The obtained results were used to determine the key amino acids that define the binding energy and type of contact between intein subunits. *In silico* substitution of five neutral amino acids in the C-part of Ssp DnaE with methionine was validated by using oligomutagenesis of a previously assembled plasmid, which was then used for *in vitro* tests with HEK293 cells. GFP reconstruction assays were used to estimate changes in trans-splicing efficiency using quantitative metrics such as the number of GFP+ cells and median fluorescence intensity as well as qualitative metrics such as microphotography and fluorescence curve analysis using live-cell microscopy. The results of the *in vitro* tests revealed significantly decreased splicing efficiency in four out of six mutant variants, with no significant differences in the other two cases. Additionally, we performed metadynamics modeling to explain how these mutations affect the molecular mechanisms of intein-intein interactions. Finally, we found a positive correlation between the structural and free energy changes in the local minima distribution and the decrease in splicing efficiency in the I151M and A162M+A165M cases. The resulting method was used with control mutations that had an experimentally confirmed positive (A168H) or negative (T198A) effect on the splicing reaction. In summary, we propose a method of free energy surface analysis in collective variables for quick and visual evaluation of mutation effects. This approach could be applied for the development of new biotechnological and gene therapy products to overcome AAV capacity limitations.

## Graphical abstract

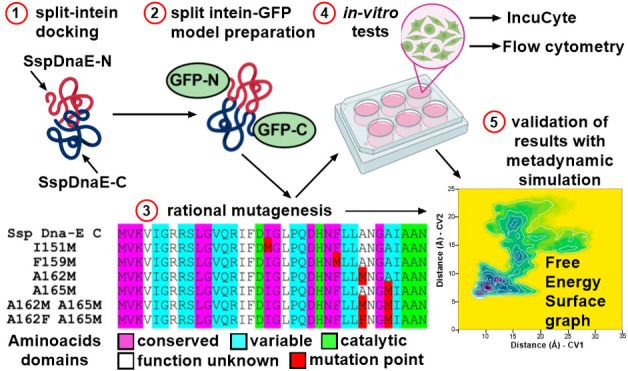

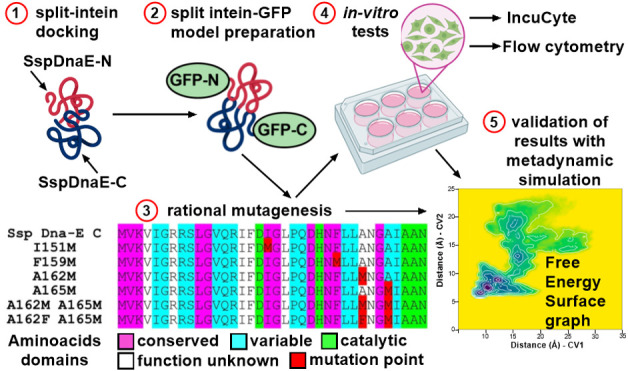

## Introduction

Inteins have become a popular and multifunctional tool in modern molecular biology, facilitating various modifications in protein engineering.^1^ This technology has been primarily applied in high-efficiency protein purification and the assembly of large protein complexes.^2^ Split inteins represent a class of supporting proteins that perform trans-splicing reactions with external proteins both *in vitro* and *in vivo*.^3^ The trans-splicing mechanism enables the joining of cleaved protein segments, which are fused with intein domains and encoded by separate DNA sequences, into a single functional protein.

This separate expression resolves several important biotechnological problems, such as assembling of toxic proteins for cell producents in the form of two inactive prespliced parts,^4^ separate delivery of genetic material to overcome the packaging capacity of viral particles,^5^ *in vivo* modification of receptors,^6^ antibody assembly,^7^ etc.

In translational medicine, the application of split inteins is justified by the perspective of creating new enhanced treatments based on a system of double recombinant adeno-associated viruses (AAV), which can increase the packaging capacity of gene therapy drugs.^8^ This approach has significantly expanded the treatment options for effective compensation of mutations in genes encoding dystrophin protein (cDNA of *DMD* gene -14 kb), Usherin (cDNA of *USH2A* gene -18.9 kb), factor VIII of blood coagulation (cDNA of *F8* gene 7 kb), and approximately a thousand other mutations related to large human genes.^9^ In addition to the separate delivery of genetic material for the replacement therapy of large genes,^8^ inteins have been used in gene editing,^10^ particularly in the assembly of adenine^11–14^ or cytosine^15–18^ base editors (ABE, CBE) and prime editors^19–21^ (PE). Current trends in the application of inteins in gene therapy are presented schematically in Figure 1.

**Figure 1:**
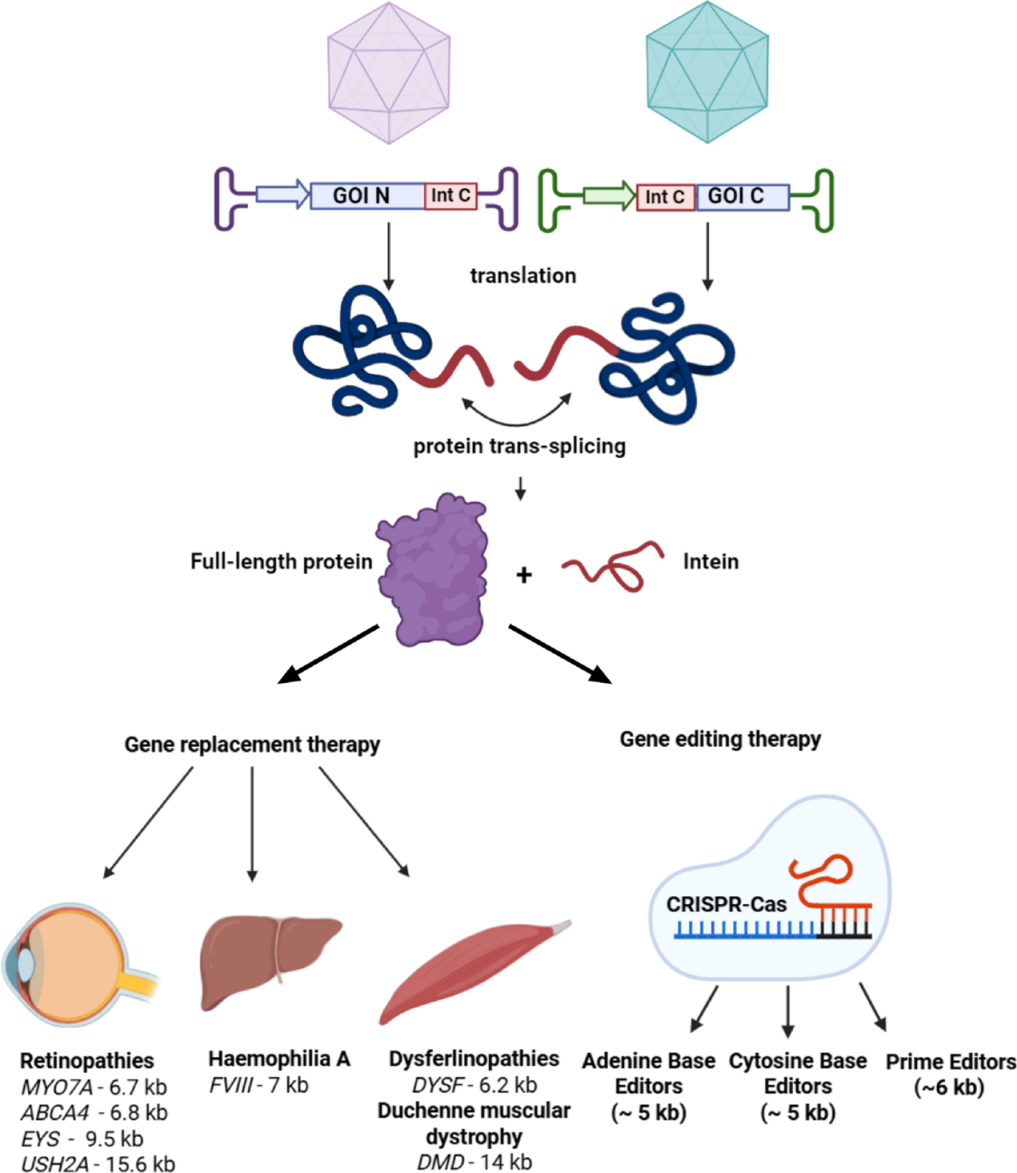
Illustration of intein trans-splicing technology for treating inherited diseases caused by mutations in large genes. Target large genes (*MYO7A, ABCA4, EYS*, etc.) are highlighted in italics with the approximate size of protein-coding DNA.

To enhance the efficiency of protein assembly in the above-mentioned applications, researchers are exploring approaches to increase the yield of protein trans-splicing reactions by modifying natural intein sequences.^22,23^ Key trends in this field include the minimization of intein sequence,^24^ detailed analysis of the structure and properties of the entire molecule^25^ and mutagenesis of individual amino acids that directly affect the trans-splicing reaction.^26^

The process of identifying and incorporating mutations has improved significantly since the first intein structure was determined using nuclear magnetic resonance (NMR). For instance, the resolution structures of the Npu DnaE intein were obtained both before and after the splicing reaction.^27^ In the same study, the authors proposed a strategy of “rational” mutagenesis to optimize the amino acid sequence of inteins to improve trans-splicing efficiency. This approach improved the affinity and stability of the intein complexes, which were accompanied by changes in the reaction kinetics between the N and C parts. Further studies on intein structures have identified the key role of the initial cysteine of the C-intein, which forms a cysteidyl-peptide thioester that subsequently undergoes an acyl shift.^28^ The investigation of intein interactions has been limited to resource-intensive experimental methods. For instance, the application of an expensive peptide synthesizer was a common approach to determine the impact of each amino acid at the N and C inteins ends and their immediate surroundings.^28,29^

Advances in structural biology methods, along with the development of molecular modeling techniques, have enabled significantly more comprehensive and detailed investigations into the structural and functional properties of biomolecules. An outstanding example of such research is the study by Stevens and colleagues,^30^ in which a synthetic consensus intein (Cfa) was engineered. Artificially constructed Cfa has 3-fold higher kinetics of the splicing reaction than the natural Npu DnaE intein. This enhanced activity was attributed to its *de novo* design, which incorporated “fast” functional domains derived from high-performance natural inteins of the DnaE group. A similar approach involving point mutations around the active center was used in the work by Eryilmaz et al.^31^

The most extensively studied split intein Ssp DnaE was selected for the analysis. This intein catalyzes the trans-splicing of α-subunit of DNA polymerase III across bacterial, archaeal and eukaryotic systems.^32^ Members of the DnaE intein group exhibit rapid trans-splicing kinetics, efficiently reconstructing protein complexes within seconds to minutes. These features make them useful tools for protein engineering. However, Ssp DnaE has been identified as one of the slowest inteins from the DnaE group, with a half-reaction time of 35 min in comparison to 63 s for Npu DnaE.^33^ Nevertheless, the number of experimentally evaluated structures for Ssp DnaE (RCSB PDB ID: 1ZD7, 1ZDE, 4GIG, and 3NZM) is considerably higher than that for other proteins in this group. This fact establishes a substantial background for the application of molecular modeling methods to predict the properties of modified inteins. The combination of these factors enables the application of rational design to identify mutagenesis points and incorporate mutations that enhance the efficiency of the trans-splicing reaction in comparison with the original Ssp DnaE pair.

The development and validation of reliable methods that would enable the modification of inteins to increase trans-splicing efficiency were set as the primary goals for this research. Ssp DnaE was used as an accessible example for modeling and experimental testing. The molecular surface of the intein Ssp DnaE was analyzed with the surface accessible to the solvent (SASA) method to identify potential mutation sites, which were incorporated into the intein nucleotide sequence. The preliminary effects of these mutations were estimated by calculating the free energy changes in affinity and stability. Following experimental validation, a retrospective analysis of molecular dynamics simulations was completed in order to explain the structural and energetic basis of the observed outcomes. The approach developed in this work can be used to modify any other intein for specific purposes.

## Methods

### Model Preparation

For computational modeling of protein–protein interactions, the resolved structures of the intein Ssp DnaE (PDB ID: 1ZDE) and the GFP protein (PDB ID: 5B61, chain D) were extracted from the PDB.^34^ Before the calculations, each model was prepared using the Protein PrepWizard module of the Schrödinger Suite 2022-4 software package.^35^ During the preparation process, water molecules were removed from the model, and the X-ray structure analysis errors were corrected using the following steps: bonding types between atoms were restored, missing hydrogen atoms and amino acids were added, charges were corrected, and protonation was adjusted. After preparation, the geometry of the protein structures was optimized using restrained minimization (Prime, Schrödinger Suite 2022-4).^36^ The following minimization settings were used: solvation model, VSGB; force field model, OPLS4;^37^ optimization method, LBFGS, with five iterations and 65 steps within one iteration. Quality control of the protein structure was performed using a Ramachandran plot.^38^

### Molecular Dynamics

Molecular dynamics simulations were performed with the Desmond program to evaluate protein stability in the solvent. The OPLS4 force field was employed for all calculations, with the following system parameters: an orthorhombic simulation box, a 15 Å buffer zone from the protein surface, and the TIP3P water model as the solvent. The aqueous solvent was designed to mimic physiological conditions (0.15 M NaCl). The system charge was neutralized by adding Na^+^ or Cl^–^ counterions. Simulation parameters: preliminary relaxation of the system was performed using a hybrid steepest descent method (15 steps) and LBFGS algorithm (2000 steps) with constraints on the dissolved component of the system, followed by energy minimization without constraints for another 2000 steps. Then, a series of short molecular dynamics simulations were performed for 12 ps in the NVT ensemble at 10 K and in the NPT ensemble at 10 K, with and without constraints on non-hydrogen atoms. A subsequent series of short molecular dynamics simulations of the system (24 ps) at 300 K in the NPT ensemble with and without constraints on non-hydrogen atoms. Main simulation: 100 ns, system integration every 2 ps, frame recording every 10 ps, temperature: 310 K, NPT ensemble. Monitored parameters: RMSD and elements of the protein’s secondary structure. Stability violations of the protein were assessed by analyzing regions with disrupted folding and abrupt changes in the RMSD magnitude.

### Protein–Protein Docking

The reconstructed and stable model of the original intein and its complex with fused GFP was used for protein–protein docking with the PIPER algorithm.^39^

A smaller protein, C-part of Ssp DnaE, was used as an interactant to reduce computational complexity during docking without specific constraints, such as repulsive or attractive potentials of amino acid groups in the presence of specific protein–protein recognition. In the docking results, 70,000 orientations were obtained, and the top 70 energetically favorable solutions were selected by clustering analysis.

Key selection criteria included pose score (an analogue of binding energy in molecular docking, considering the interaction energy of contacting amino acids, kcal/mol) and pose energy (the energy of the entire protein–protein complex, accounting for potential strain that may arise outside the interface of the protein–protein interaction, kcal/mol).

### Protein Surface Analysis

Analysis of the molecular surface of intein was used to identify the primary and secondary amino acids that form the protein–protein interface. For surface analysis, the intein with parts of GFP was split into two separate chains. The “heavier” fragment with the N-part of the intein was used in the calculations. The method was based on calculating the molecular surface accessible to the solvent (SASA) and projecting the potentials of free energy (Δ*G*) of non-bonded interactions between atoms. The lipophilicity characteristics and electrostatic potentials were calculated, and the reactive amino acids were identified based on the solvent accessibility of their side chains.

### Molecular Metadynamics

The resulting molecular structures were used to investigate the effects of mutagenesis on the conformation of the studied proteins. The method of molecular metadynamics was used to evaluate changes in the free energy of the system (Δ*G*) and to associate its values with conformational rearrangements in the coordinates of the selected collective variables (CVs).

The following parameters were used for the metadynamic calculations: all investigated protein models were placed in an orthorhombic region with a buffer distance of 15 Å from the protein surface. A molecular system simulating a physiological solution (0.15 M NaCl) based on the TIP3P water model was used as the solvent. The charge of the proteins was neutralized by the addition of appropriate counterions (Na^+^ and Cl^–^). The OPLS4 force field was used for the calculations.

The following collective variables were used to calculate the free energy surface

CV1:B:D150−C170↔A:V184−L224

CV2:B:D150−C170↔A:G134−V184

The centroids of the amino acid groups were chosen to represent the center of mass of the most stable elements of the protein structure. The sequence between centroids is labile and undergoes conformational changes during the splicing process. Therefore, the analysis of free energy surfaces revealed changes in the geometry of intein during the splicing reaction.

The total simulation time for metadynamics was 200 ns with frame recording at 10 ps and energy recording at 2 ps. The temperature was 310 K, pressure was 1 atm (1.013 bar), biasing potential was 0.03 kcal/mol, and potential input interval was 0.09 ps. Ensemble - NPT, with mandatory preliminary relaxation of the entire system, was used.

### Plasmid Cloning

Basic plasmids for this study were obtained during our previous work.^40^ Mutagenesis of Ssp DnaE C was performed during the PCR with the Tersus Plus PCR kit (PK221, Evrogen, Russia) and primers containing intein nucleotide sequences with single or double mutations (Table S1). A typical PCR contained 4 μL of 5× Tersus Red buffer, 0.5 μL of 10 mM dNTP mix, 0.5 μL of 50× Tersus polymerase mix, 2 μL of 10 pM oligonucleotide mix, 12 μL of dH_2_O, and 1 μL of DNA template. The following amplification protocol was used for the PCR on a T100 thermal cycler (1861096, BioRad, United States): initial denaturation at 95 °C for 5 min, followed by 30 cycles of denaturation at 95 °C for 10 s, annealing at 60 °C for 20 s, and extension at 72 °C for 30–60 s, depending on the size of the PCR product. The amplified PCR products were purified with the CleanUp Mini Kit (BC023L, Evrogen, Russia) according to the manufacturer’s protocol. Purified constructs were cloned via *BamH*I and *Hind*III sites into the pAAV-CMV-MCS expression vector from the AAV5 CMV Expression System Kit (VPK-410-SER5, Cell Biolabs, United States). Positive bacterial clones were verified by PCR and Sanger sequencing (ABI 3500, Thermo Fisher Scientific Inc., United States).

### Transfection of HEK293

The adherent HEK293 cell line (85120602, ECACC, 2024) and cationic polymer polyethylenimine PEI-MAX (24765, Polysciences Inc., United States) were used for transfection. The transfection mixture was prepared according to the following protocol: 2.5 μg PEI (PEI/DNA molar ratio of 5:1) was dissolved in high glucose DMEM (D5648, Sigma-Aldrich, Germany) and mixed with 0.25 μg of each plasmid of inten’s pair (0.5 μg of total pDNA per well). After 12 min of incubation for the generation of the PEI–DNA complex, the transfection mixture was added to 2×10^5^ trypsinized adherent cells in DMEM supplemented with 10% FBS (S181H-500, Biowest, France). The suspension was gently mixed, and the transfected cells were seeded in a 48-well plate in three biological replicates per sample. After 24 h, the medium containing the transfection reagents was replaced with 0.4 mL of fresh DMEM containing 5% FBS for a further 48-h incubation.

### Live-Cell Microscopy

Fluorescence microscopy scanning and analysis were performed with IncuCyte S3 (Sartorius AG, Germany). The instrument was programmed with a scanning schedule of every 2 h in two optical channels (brightfield and green). The instrument’s built-in software was used for qualitative and quantitative analysis of fluorescence for each scan (number of GFP+ objects per image) and in the dynamics of fluorescence accumulation.

### Flow Cytometry

After 48 h of incubation, cells were trypsinized for flow cytometry analysis. For sample preparation, the cells were washed twice with 500 μL of PBS and then resuspended in 250 μL chilled FACS buffer (1× PBS, 5% FBS, 1 mM EDTA). Data were collected using a CytoFlex B2-R2-V0 flow cytometer (A00-1-1102, Beckman Coulter, United States) and analyzed with CytExpert v1.2 software to detect GFP+ live cells. Live cells were gated based on the population distribution by FSC-A and SSC-A. Single cells were gated based on population distribution using the FSC-A/FSC-H plot, and the percentage of GFP+ cells was determined by comparison with autofluorescence of negative control cells without plasmid transfection. Further statistical analyses for normality of sample distribution and confidence intervals for rejection of the null hypothesis were performed using GraphPad Prism 9.3.1 (one-way ANOVA with Sidak’s test for multiple comparisons). Results are presented as the mean ± standard deviation of 3 biological replicates. Confidence intervals are as follows: significant (*) *p*-value <0.05, (**) *p*-value <0.01, (***) *p*-value <0.001, (****) *p*-value <0.0001; nonsignificant (ns) *p*-value >0.05.

## Results

### Model Preparation for Calculations

The consensus model of intein annealed with GFP split at 69 amino acids was constructed from resolved structures of Ssp DnaE intein precursor (1ZDE) and GFP (chain D in model 5B61). The selection of the trans-splicing site for GFP disruption was previously experimentally confirmed and discussed in our previous study.^40^

The stability of the resulting intein model with GFP was checked by using molecular dynamics. The calculations have confirmed the asymptotic nature of the RMSD curve (Figure 2), indicating that the protein was stable and did not undergo any significant structural changes.

**Figure 2:**
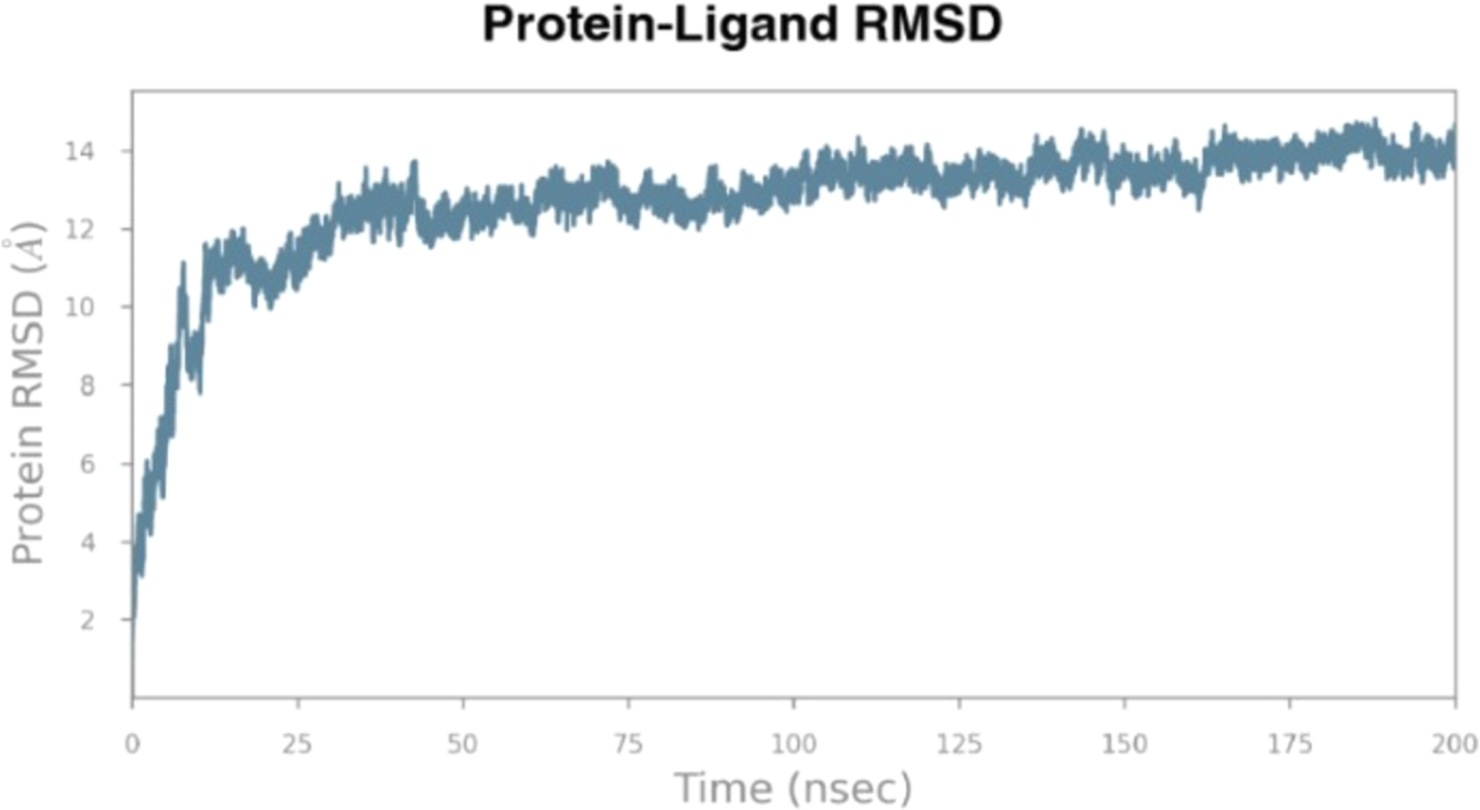
Graph of root-mean-square deviation (RMSD) changes during 200 ns of molecular dynamics simulation with the GFP-intein resulting model. The asymptotic curve of the RMSD has confirmed the stability of the resulting intein model.

Protein–protein docking was used to verify the ability of the obtained composite structure to restore the original intein complex. Initially, the Ssp DnaE intein model (PDB ID 1ZDE) was manually divided into N and C-terminal parts based on the amino acid sequence from the Intein Database.^41^

Subsequently, limited minimization of the protein structure was performed to relax the side chains from conformational changes induced by the interactant and normalize the hydrogen bonding network. The separated parts of the intein were subjected to protein–protein docking (Figure 3). The evaluation function metrics and clustering indicated that the most energetically favorable configuration corresponds to the original protein–protein complex (cluster size = 183, pose score = −773.41 kcal/mol, pose energy = −2887.77 kcal/mol). Thus, protein–protein docking confirmed that the constructed Ssp DnaE intein was restored to the original protein–protein complex (Figure 3 A, B).

**Figure 3:**
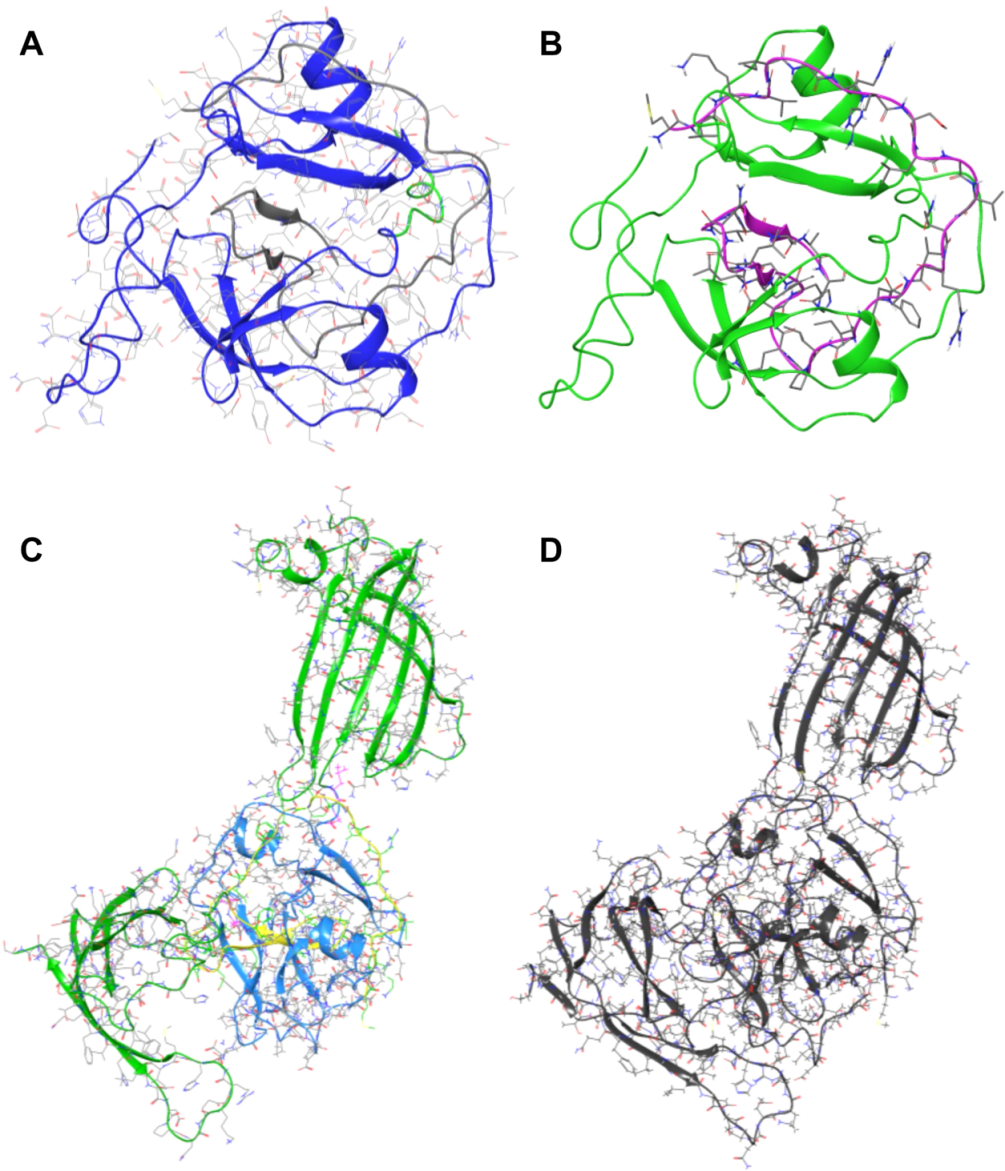
Reconstructed protein models: (A) original intein complex from 1ZDE structure; (B) the result of protein–protein docking evaluation of N- and C-terminal parts of Ssp DnaE intein; (C) complex of intein fused with GFP split at 69/70 amino acid; (D) the resulting consensus model after protein–protein docking of N and C-terminal parts of intein Ssp DnaE fused with GFP.

Similarly, protein–protein docking was performed with an identical protocol to obtain a model of the intein fused with GFP split into two parts. The results of the calculations are listed in Table 1. Variant 2 was selected as the best solution in the protein–protein docking result, with the highest similarity to the original protein–protein complex (Figure 3C,D). Other variants also reproduce the original complex, but with lower clusterization resolution.

**Table 1:**
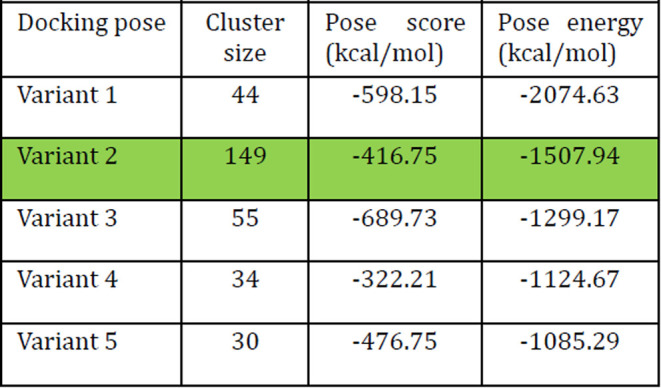
Results of the Top 5 Solutions after Protein–Protein Docking^a^

a The best solution is highlighted in green.

The calculations at this stage confirmed the thermodynamic stability of the obtained construct. Models with errors would either be energetically unfavorable or produce incorrect conformations.

### Rational Design Mutagenesis of Ssp DnaE

In the next stage, an analysis of the active surface in the intein structure was performed to identify the major and minor amino acids involved in the formation of the protein–protein complex. Initially, the N/C intein fused with GFP was separated into two distinct chains. A larger fragment containing the N-part of the intein was used for further molecular surface analysis.

For the selected structure, the molecular surface was calculated and mapped for potential non-bonded interactions, which were divided into three general types: lipophilic, positive electrostatic, and negative electrostatic. The maximum possible intermolecular interaction potential (analogous to the Gibbs free energy in kcal/mol) was calculated for each amino acid forming the surface segment. This calculation considers the available molecular surface area of the amino acid side chain and classifies it as lipophilic, polar, or charged.

Mutations were selected by evaluating the magnitudes of intermolecular interaction potentials and the types of amino acids from the C-part of the intein that interact with the mapped surface of the N-intein. These amino acids and their intermolecular interaction potentials are listed in Table 2

**Table 2:**
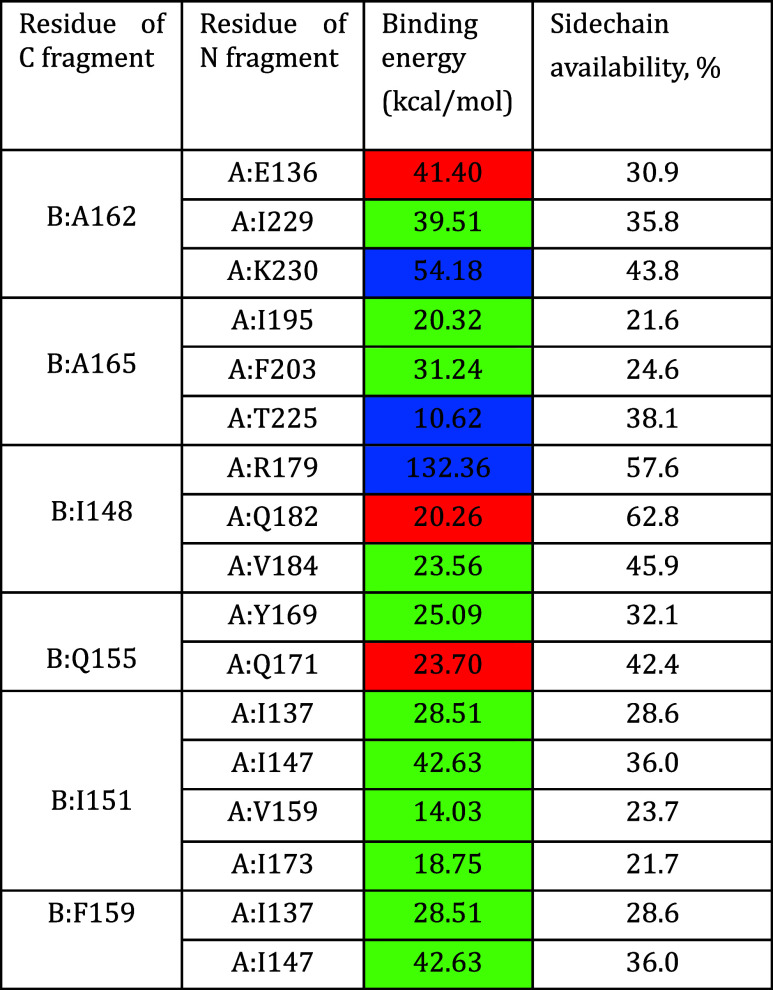
Binding Potentials of the Interacting Amino Acids in the Intein Structure^a^

a Contact type: red—negative electrostatic potential; blue—positive electrostatic potential; and green—lipophilic.

The pool of possible point mutations was determined by the values of the potential intermolecular interactions. For example, A162 was estimated to be optimal for the replacement of alanine (a lipophilic amino acid) with a polar or more accessible side-chain amino acid with a negative charge localization. The complete panel of possible point mutations is listed in Table 3.

**Table 3:** Variety of Mutations in the C-Part of Ssp DnaE Intein after Evaluation of the Binding Interaction Potentials

residue of the intein C-part	point mutation variants
B/I148	Y, N, Q, H, S, T
B/I151	A, C, G, L, M, F, P, W, V
B/Q155	R, I, F, A, N, D, C, E, G, L, K, M, P, S, T, W, Y, V
B/F159	M, A, C, G, I, L, P, W, V
B/A162	F, C, G, I, L, M, P, W, V
B/A165	V, W, P, F, M, L, I, G, C

The number of mutations was limited to only single and double simultaneous mutations by exponentially increasing the complexity of the calculations. Initially, the mutation effect was predicted by changing the Gibbs free energy in affinity (ΔΔ*G*) and stability (ΔStability) parameters within the protein–protein complex. In addition, conformational rearrangements of amino acids induced by the introduced mutation were calculated within 7 Å around the mutated amino acids. Mutant models were selected by optimal combination of ΔΔ*G* and Δstability parameters and selected variants are listed in Table 4.

**Table 4:** Evaluation of Ssp DnaE C Part Mutations Influence on the Trans-Splicing Efficiency^a^

	calculated free energy changes		experimental data from 48-h post-transfected HEK 293		
samples	affinity ΔΔ*G* dG(kcal/mol)	stability Δstability dG(kcal/mol)	mean %GFP+	mean MFI	mean number of GFP+ objects per image
pGFP	-	-	93,7	236959	5711.78
A165M	–18.92	0.74	64,0	11132	1099
SSP N+C	-	-	62,3	9947	1775
A162M	–3.25	0.92	59,4	6250	992
F159M	–4.61	–2.28	43,4	1609	50
A162F+A165M	–31.73	–26.29	41,6	1416	129
A162M+A165M	–22.92	–14.04	26,2	1042	22
I151M	–14.00	–10.28	16,4	671	1.5
HEK293	-	-	0,3	450	0.1

a Experimental results after transfection of HEK293 were compared with the calculations of free energy changes in affinity and stability.

### Experimental Verification of the Effects of Mutations on the Trans-Splicing Efficiency in HEK293 Cells

Six selected mutations (Table 4) with the highest changes in ΔΔ*G* and Δstability parameters were used for the gene-engineering plasmid modification. Original plasmids for estimation of changes in the trans-splicing efficiency by yield of the intein-mediated GFP reconstruction were obtained in a previous article.^40^ Mutagenesis primers (Table S1) were used for overlapping PCR to modify the nucleotide sequences corresponding to the selected amino acids.

Six mutant Ssp DnaE C fragments were obtained by PCR and used for cloning. Non-modified pairs of the Ssp DnaE-N/C-GFP plasmids were used to compare the effects of Ssp DnaE-C modifications. pGFP plasmid was used to compare the direct expression of full-length GFP with intein-mediated reconstruction of GFP divided at 69/70 split site. Untransfected HEK293 cells were used as a negative control for cytometry gating of GFP+ cells by values of measured autofluorescence. The resulting experimental data are presented in Table 4 and Figure 4.

**Figure 4:**
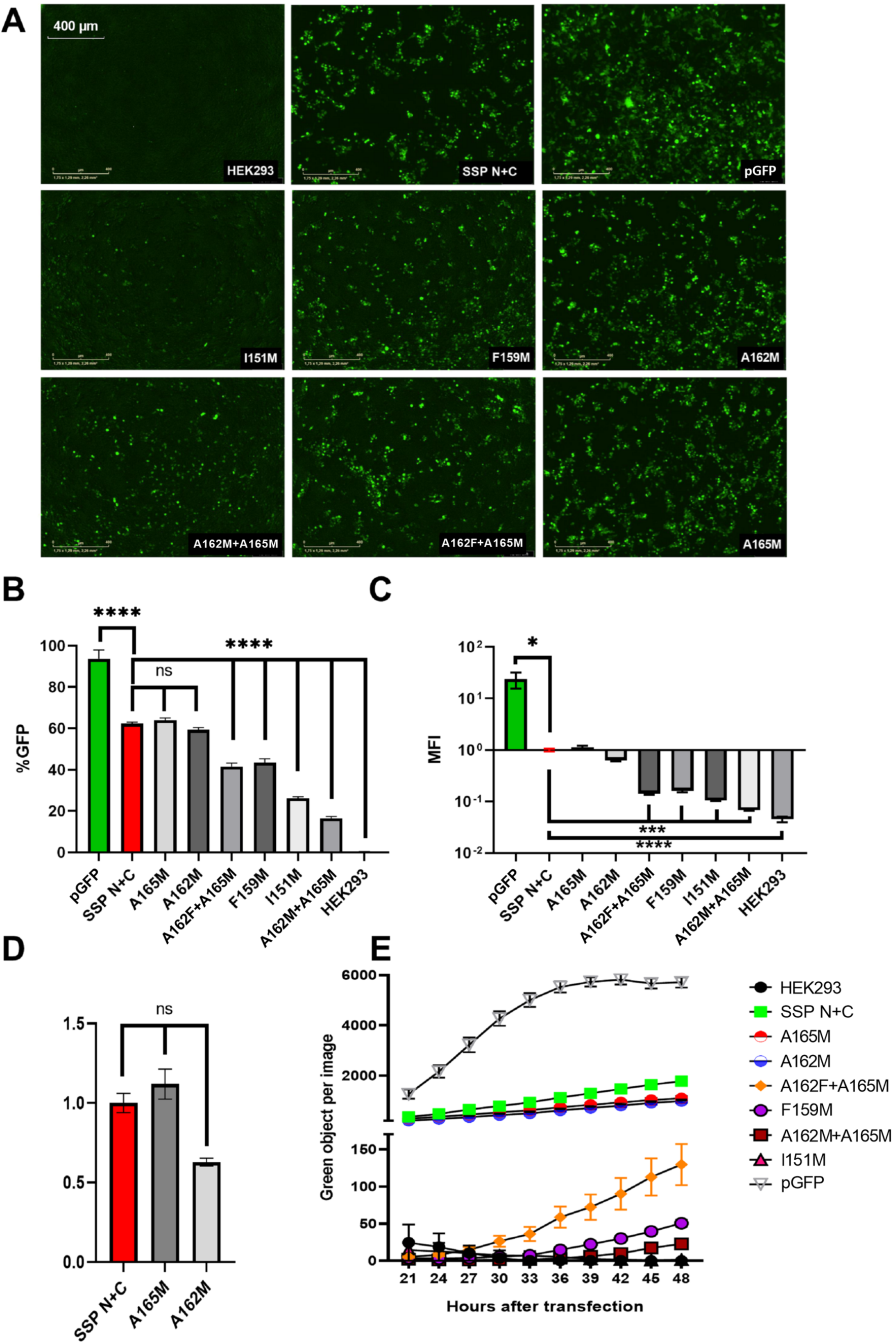
Experimental validation of the predicted mutagenesis effect on trans-splicing efficiency in combination of SspN-GFP and versions of SspC-GFP (short name on plot SSP N+C): (A) fluorescence microphotographs of 48 h post-transfected HEK293 cells captured by the IncuCyte S3 with parameters: six images per well with 10× magnification and 300 ms of GFP exposure. (B) Flow cytometry analysis of GFP+ cells population, (C) flow cytometry analysis of MFI values normalized to the fluorescence of the Ssp N+C sample in 10-base logarithmic scale, (D) enlarged version of MFI values normalized to the fluorescence of the Ssp N+C sample, (E) kinetic curves of green objects per image accumulation from cells during 24 h incubation in IncuCyte S3. (*) *p*-value <0.05, (**) *p*-value <0.01, (***) *p*-value <0.001, and­(****) *p*-value <0.0001.

The samples were arranged by the percentage of GFP+ cells and MFI (Table 4). The A165M sample with simultaneous positive changes in stability (Δstability) and negative changes in affinity (ΔΔ*G*) had slightly higher %GFP+ and MFI values compared to the original pair Ssp N+C. Otherwise, simultaneous changes in affinity and stability resulted in a 20% decrease in efficiency for the double mutation A162F + A165M and a 35% decrease for A162M + A165M. The difference between the experimental results and calculations based on energy changes with the SASA method indicated that a different approach should be used to predict the effect of mutations at the calculation stage. Fluorescence analysis of micrographs (Figure 4A) demonstrated that the single mutation I151M and double mutation A162M + A165M resulted in a significant decrease in trans-splicing efficiency. Notably, single mutations at the same positions, A162M and A165M, led to the opposite effect, which is clearly illustrated in Figure 4B–C. Kinetic curve analysis (Figure 4E) was used to cluster samples into two groups with amplitude similar to that of the original pair (SspN+SspC, A162M, and A165M) and decreased amplitude in all other samples.

The population analysis of GFP+ cells (Figure 4B) was used to cluster the samples more precisely into three groups: the group with insignificant efficiency changes in samples A162M and A165M, the group with significant efficiency decrease up to 20% in samples A162F + A165M and F159M, and the group with efficiency decrease of more than 30% in A162M + A165M and I151M. MFI analysis with fluorescence normalized to SspN+SspC was plotted on a 10-base logarithmic scale (Figure 4C). Intergroup differences between Ssp N+C, A162M, and A165 M were not significant. Significant differences were estimated in the samples A162F+A165M, F159M, and I151M within 10 fold change of magnitude from the control. A higher level of significance was obtained in samples A162F + A165M, and the intensity was the same as that of the negative control. Additional subplot (Figure 4D) with fluorescence normalized to Ssp N+C was plotted without a logarithmic scale for a more detailed estimation. Despite the absence of significant differences, a positive effect of the A165M mutation and a negative effect of the A162M mutation were observed.

Summary analysis of the experimental data revealed a negative effect for mutations in I151M, F159M, A162M + A165M, and A162F + A165M and a neutral or slightly positive effect for A162M and A165M. These results were used to analyze protein–protein interactions with molecular metadynamic modeling.

### Molecular Metadynamics Analysis of Mutated Protein Structures

The method of molecular metadynamics was used to analyze and explain the experimental results obtained in terms of molecular dynamics. This method was used as an alternative to predict the effects of mutagenesis by analyzing the free energy changes in the system in terms of selected collective variables. The I151M and A165M mutations were used to explain the role of single mutations at different structural points of intein. Mutations A165M and A162M + A165M were used to explain the negative effects of double mutations with methionines close to each other. The selected mutations demonstrated a contrast effect on trans-splicing efficiency and could be used for a better understanding of intein’s structural features.

Free energy surfaces were evaluated for selected mutations in the coordinates of the collective variables (Figure 5). The obtained surfaces could explain mutations effects by the rearrangement of local energy minima, which determine the lability and flexibility of intein molecules and kinetics of the splicing process. For instance, the partitioning of free energy into multiple oriented minima indicates changes in molecular interactions (Figures 5A,C). *In vitro* tests in the case of A165M did not show a significant change in efficiency with the cell assay; therefore, other methods should be employed for further investigation. Otherwise, the free energy concentration at a single major minimum was correlated with a decrease in splicing efficiency (Figures 5B,D). This common trend has introduced a new insight for the primary prediction approach to estimate the mutagenesis effect on trans-splicing by the analysis of local free energy distribution and estimation of energy barrier height between them.

**Figure 5:**
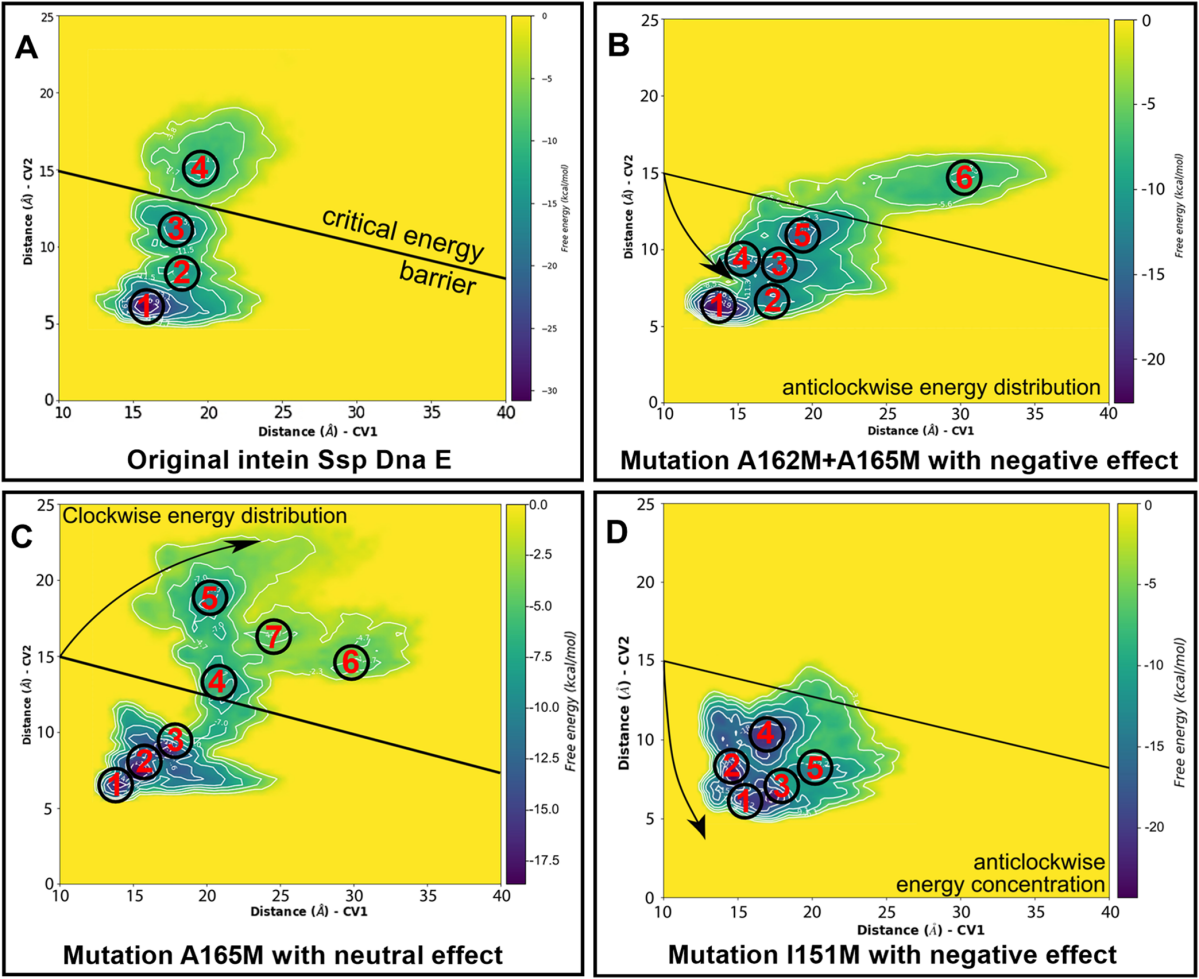
Free energy surfaces obtained from 200 ns metadynamic calculations of Ssp DnaE intein fused with GFP: (A) without mutations, (B) double A162M+A165M mutation, (C) single A165M mutation, and (D) single I151M mutation. Local minima of free energy are numbered in circles, and colors represent the energy value. Individual images with fitted axes are placed on Figures S1–S4.

The obtained metadynamic analysis results (Table S2–S5) were used to illustrate the energy distribution with surfaces (Figure 5). Similar scales of collective variable coordinates enable the estimation of the clockwise and anticlockwise directions of the free energy distribution. A borderline was placed on plots between points (0;15) and (40;8) in CV1–CV2 coordinates to separate minima in the left corner with coordinates that describe the interaction of C-part centroids with the N-terminal part centroids. This visual implementation enables quick estimation of the most probable effect of mutations on splicing efficiency changes. A detailed explanation of each mutation effect will be discussed further.

Primarily, the A165M mutation with a clockwise free energy distribution has a positive effect on the splicing reaction. Additionally, A165M was characterized by an increased number of local minima shifted to the right by the CV1 coordinates with a decreased height of the energy barrier compared to the original intein. The most evident rearrangement occurred by shifting the barrier value from +18.75 kcal/mol between 1 and 2 points in the non-mutated intein to +10.93 kcal/mol between 3 and 4 points in A165M (Table S3). This fact has made energy clusterization more distinguishable by coordinates and allowed us to draw a line on plots that defines the direction of the energy distribution. The decrease in the energy barrier, along with the increasing value of the common free energy (12.71 kcal/mol), has enabled the expansion of energetically permissible intein conformations. The summary effect of the A165M mutation can be characterized as an increase in conformational mobility with a higher number of energy minima and lower energy barriers, resulting in reaction-competent states with lower energy transfer costs.

Secondly, mutations with negative effects, I151M and A162M + A165M, were characterized by an anticlockwise energy distribution. In the case of the double mutation A162M + A165M, the number of minima has increased from four in the original structure to six. This rearrangement of the minima increases the conformational mobility of the molecules. However, the double mutations A162M + A165M and A165M had almost equal numbers of minima, but most of them were placed on the left side of the CV1 coordinate. The inhibitory effect of the second methionine mutation was indicated by the minima concentration at the position of the initial state, with the higher barrier between 1 and 2 points in A165M with+4.24 kcal/mol (Table S3) and A162M + A165M with +10.96 kcal/mol (Table S4).

This fact has increased cost of energy transfer, and collapsed intein's viable conformations. In addition, structural analysis has revealed evidence of changes in the profile of hydrophobic contacts within the intein core around amino acids A/L189, A/V194, A/I195, and A/P144 (Figure S7).

A similar effect was observed for the I151M mutation. Additional hydrophobic contacts became available with A/I137/147, A/V159, and B/F159 due to the longer chain length (5.51Å M vs 3.87Å I). Essentially, the I151M mutation has stimulated hydrophobic contacts between chains A and B of the intein while maintaining contacts with B/L153 (Figure S8). This region has originally formed a “leucine zipper” that was stabilized with an I151M mutation. The conformational mobility remained original with the same number of minima, mostly at the initial positions. Suddenly, the mutation has caused a decrease in each energy barrier with a simultaneous increase in the depth of each minimum.

In summary, the I151M mutation has a dual effect. On the one hand, energy barriers were significantly lower than those in other cases, and conformations were available for expansions and transitions. The concentration of free energy in the four deep minima and the formation of additional hydrophobic contacts made all conformations energetically unfavorable for the molecular system. The resulting overstabilization of the hydrophobic core of the intein molecule makes trans-splicing energetically unaffordable.

These examples have demonstrated that free energy analysis could indirectly highlight the structural anomalies caused by important conformational changes, which should be additionally observed to accept the final decision regarding the predicted mutation effect.

### Molecular Metadynamics of Control Mutations

The results from the previous section were applied to the metadynamic analysis for two control mutations, whose effects had been previously experimentally estimated.

Mutation T198A completely blocked the trans-splicing reaction^42^ and mutation A168H increased molecular stability and splicing efficiency.^43,44^ These facts were used to incorporate these mutations into the obtained model of GFP fused with intein Ssp DnaE. The results of the metadynamic simulation for this model are illustrated (Figure 6).

**Figure 6:**
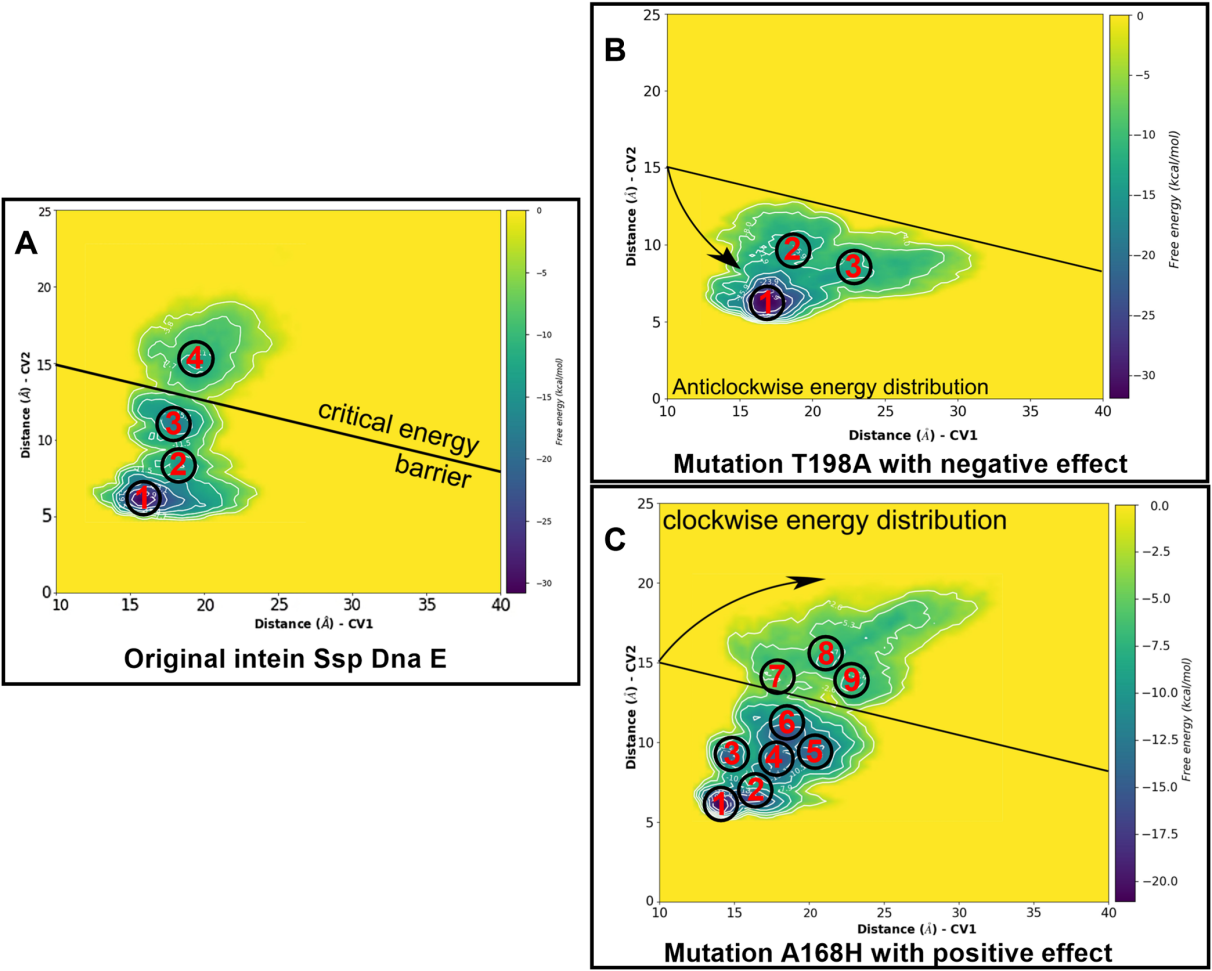
Free energy surfaces obtained from 200 ns metadynamic calculations of DnaE-fused Ssp intein with GFP: (A) without mutations, (B) T198A mutation, and (C) A168H mutation. Local minima of free energy are numbered in circles, and colors represent energy value. Individual images with fitted axes are placed on Figures S5–S6.

The simulation results have supported the experimental data in the context of the free energy and structural changes in the model. The threonine-alanine replacement has disrupted the hydrogen bond network between D200 and Q182, where T198 was acting as a bridge, realizing transient contacts (Figures S9 and S10). From the position of energy changes, the T198A mutation forms deep energy minima 1 (Figure 6) with a high energy barrier for transitions to local minima 2 and 3 (Table S6). At the same time, the free energy of the system is even lower than that of intein without mutations (Figure 6, Table S2).

In the structure without mutations, there are 4 energy minima, while with the T198A mutation, the fourth minimum disappears from the initial coordinates.

As a result, we observe the formation of a deep energy minimum at point 1, with a high energy barrier for the transition to local minima 2 and 3.

The opposite effect was observed for the A168H mutation. Surface analysis (Figure 6C) revealed an increase in the number of energy minima from the original four to nine with a shift of the CV1 coordinates, which improved the mobility of the molecule. The energy barriers in this case were reduced to almost zero between minima 4 and 5, and between minima 5 and 6 (Table S7). In addition, an overall increase of free energy (+11.13 kcal/mol) made the intein system more flexible and labile. This positive effect should provide active sites for the splicing reaction and increase its efficiency. Structural changes after the A168H mutation with the formation of π–cation contacts with A/R202 and hydrogen bonds with B/D171 and B/N 158. These intermolecular interactions have stabilized the core with a powerful source of intramolecular contacts during conformational rearrangement along the splicing reaction (Figure S11).

In summary, the results obtained with the metadynamic simulation were validated by experiments in this study and additionally tested with control mutations. The results in the previous section were confirmed, and the combination of metadynamics and structural analysis could be used as a predictive tool for further modification of the intein structure using point mutations.

## Discussion

This study was done as a continuation of our previous research.^40^ The development and validation of a site-directed mutagenesis method were the primary goals of this study. The obtained metadynamic method of analysis could be used for the estimation of mutagenesis of any inteins with correction to structure analysis.

Particularly, modification of slow Ssp DnaE intein was performed with an insignificant experimental increase of trans-splicing efficiency in case A165M and a considerable decrease in other cases. However, analysis of free energy changes has revealed trends in structural changes that are essential for trans-splicing efficiency. Furthermore, this trend was replicated with results from control mutations that supported the findings from this and previous studies.^42–44^

The implementation of a metadynamic analysis to estimate the mutagenic effect is a key feature of this study. Previously, this method has been used to estimate the inhibitory effect of molecules^45^ and the impact of each amino acid in exteins.^31^ Free energy distribution analysis in collective variables was performed in inteins to predict the possible initial confirmation for the first time. In previous studies, RMSD trajectories, covariance matrices of backbone Cα atom motion,^46^ and principal component analysis on Cα coordinates^47^ were used to describe the effect of mutations implemented with the molecular dynamics method. The implemented method was double-checked with new and control mutations, which made this approach more reliable than the primary estimation of changes in stability and affinity.

New mutation points were obtained from the active surface analysis of protein models, which has been widely used to analyze protein–ligand or protein–protein interactions.^48^ This approach was also used for the first time in intein mutagenesis. Geometric structure analysis with Ramachandran plots was alternatively used to identify structural features that could significantly change the splicing yield.^42^

Experimental screening of amino acids with more favorable chemical properties is the most common method of directed mutagenesis.^43^ The structural analysis of the static and dynamic changes supported the experimental data with more detailed information on intermolecular mechanics.^44,49^ Notably, all listed chemical methods of intein rational modification were able to perform single precise mutations in the conservative and catalytic domains to significantly change the trans-splicing efficiency.

“Irrational” methods of intein modification have also been used with computational methods in genetics. Consensus analysis of the amino acid sequence with annotation of functional domains has enabled simultaneous manipulations with multiple mutations. Cfa intein was assembled with the fast domain of DnaE inteins after alignment and annotation of 73 sequences. The resulting intein has demonstrated 3 times faster kinetics than NpuDnaE, which has the highest yield and kinetics in the natural intein group.^30^ The major drawback of this modification method was revealed in the experimental application of Cfa for trans-splicing reconstruction of large protein complexes.^50^ Suddenly, the yield of target protein splicing was significantly higher for NpuDnaE intein than for Cfa and Ssp DnaE. This unexpected result was obtained as a consequence of the uncovered features of the artificial intein, which has individual structural features at the protein level. However, structural analysis and targeted precise modifications of the C-part of the Cfa intein have already been performed to increase the yield in the case of protein purification.^22^

Implemented point modification of the C-part of Ssp DnaE has mostly decreased the yield of the trans-splicing reaction but has also revealed structural features. A series of single-point mutations with changes from neural amino acids to methionine in different domains of intein could be used to better understand the role of individual residues in the structure of intein. The general trend emerged from the observation that trans-splicing efficiency decreases in the direction from the peripheral catalytic part to the intein’s core with the mutations A165M, A162M, F159M, and I151M. In the peripheral part at A165 and A162, the efficiency changes slightly in the percentage of GFP+ cells. This flexible domain was bound by F159 with a significant decrease in dozens of percent of GFP+ cells. The core domain with I151 could be identified with a drastic decrease in GFP+ cells, similar to the negative control. This fact was additionally explained by the analysis of the structural changes in each case. For instance, I151M has formed an increased number of lipophilic contacts in the core with N- and C-parts, which has increased the absolute energy minimum and overstabilized the C-part molecule.

The same negative effect was obtained with the control T198A mutation in the case of destabilization of the N-part. This mutation was associated with the most conserved tyrosine located in the catalytic and geometrical centers of the N-part of the intein. Two mutations in this core position with opposite effects were tested after extraction of intein structural features based on Ramachandran plot analysis.^42^ T198S mutation demonstrated a neutral effect on splicing with insignificant changes in geometry, whereas T198A destabilized the structure and completely blocked the response. These experimental data were supported by the calculations in this study. The docking results illustrated that amino acid Y198 formed a bridging bond between D200 and Q182 in the initial state of intein. Replacement of tyrosine with alanine has disrupted these hydrogen bonds and destabilized the molecule at the core of the N-part of the intein. Free energy surface analysis reflected these structural changes with a decreased number of energy minima and an increased height of the energy barriers. Similar results were obtained in the case of I151M. The conclusion regarding the negative influence of increased energy barriers on the splicing reaction is consistent with previous research.^42^

Unexpected results were obtained from the analysis of experimental data with the double mutations A162F + A165M and A162M + A165M. Firstly, these results have disproved the primary method of prediction of mutation effects based on energy changes with affinity (−ΔΔ*G*) and stability (ΔStability) parameters. Secondly, structural analysis replaced this stage after the discovery of a significant decrease in splicing efficiency in the case of simulated double methionine mutations in A162M + A165M. Notably, similar single mutations at similar positions in the cases of A162M and A165M did not demonstrate such effects. This fact suggests that double methionines have formed contact with each other, but structural analysis has demonstrated only destabilization of the C-part with the extensive form of each methionine molecule. Moreover, surface analysis has allowed a visual comparison of A162M + A165M with A165M and has outlined the effect of A162M in changing the number of lipophilic contacts with the same number of energy minima. Additionally, the formation of lipophilic contacts has shifted the coordinates of the minima to the CV2 axes and increased the height of the energy barriers. In summary, the overall mobility of the molecule has been increased, but structural changes have prevented reactions from favorable splicing interactions.

In the case of the experimentally neutral effect of the A165M mutation, both metadynamics and structural analysis have investigated mostly the positive effects of alanine to methionine substitution. The map of lipophilic contacts between the N- and C-portions was modified by the formation of lipophilic contacts with F203, I195, L223,/189 and disruption of a quartet between A/A197, A/I195/213, and B/L161. The location of the mutation at the boundary between two structured regions (β-sheets transitioning into loops) has affected the conformational rearrangement due to steric constraints that change the mechanics of the conformational transition. The immediate proximity of the mutations to the peripheral catalytic center at the end of the C-part of the intein has provided a positive effect in the case of A168H. The substitution of alanine with histidine has created powerful π-cationic interactions between arginine at position 202 and hydrogen bonds between histidine and aspartic acid at position 158 and aspartic acid at position 171. These interactions have stabilized the intein core during conformational rearrangement for splicing. Metadynamic calculations have revealed the formation of additional energy minima with low energy barriers, which made the molecular system more conformationally labile and promoted an efficient trans-splicing reaction. In both cases A165M and A168H, the replacement of alanine with structurally more extended amino acids has formed additional contacts that have stabilized the catalytic C-part center and further accelerated the cyclization of aspartate in the trans-splicing reaction.

Stabilization of inteins has been used “as the simplest approach for rational engineering of proteins”.^51^ Other chemical approaches have been used for the targeted deletion of several amino acids by NMR analysis to increase the cleavage of the N-part to promote the trans-splicing reaction.^52^ Another modification method based on NMR analysis was used to enable the splicing reaction in a 2 M NaCl solution by replacing positively charged amino acids with neutral ones.^26^ Biological approaches have also been used to find genetically variable domains by screening all 20 amino acids with oligonucleotide-directed mutagenesis.^53^ As a result, dozens of DNA libraries must be tested experimentally with random results, mostly without any chemical explanation.

The most effective biological approach is the discovery of new shorter natural inteins from the results of genomic data analysis. For instance, gp41 intein was discovered as a result of massive metagenomic data analysis and currently has the faster kinetics than Npu DnaE by 10 times.^54^ However, its resolved structure was only recently obtained^55^ and its features have not yet been fully studied. In particular, the discovery of new inteins did not stop the search for modifications and mutations of newly discovered molecules.^56^ This problem and the method of solution have already been discussed in the study of increasing reaction yields,^57^ which is particularly important in protein purification.^22^ Therefore, the development of new approaches for rational mutagenesis of inteins, optimized for each specific purposes, would also be relevant for the discovery of new inteins. The method of metadynamic simulation would significantly accelerate the process of hypothesis testing.

## Conclusions

In this discussion, the most relevant methods of intein modification were listed. The main trend was outlined in the discovery of new short inteins and the modification of their structures. The most common modification was identified as stabilization of the C-terminal catalytic site of inteins to accelerate cyclization in the splicing reaction. Other findings were also obtained from the experimental data.

First, the application of active site analysis should be modified with corrections for genetic analysis of conserved and variable positions. Interactant estimation should be used for further selection of amino acids, but the selection method should be modified with structural analysis. Domain functional analysis should be used to estimate the parameters that would be optimized for a particular purposes. For instance, in the case of trans-splicing efficiency, optimization of the C-terminal catalytic site should be stabilized, and analysis of energy changes in Δstability was applicable. In other cases, this method was not applicable due to the structural features.

Second, molecular metadynamics methods were used to predict the mutation effects. The main feature of this method was revealed with the analysis of free energy surfaces in collective variables, which could indicate significant structural rearrangements by coordinates and the type of minima allocation and grouping. The concentration of minima in a cluster with high values of energy and barriers has indicated structural changes that could reduce conformational lability. Otherwise, the widespread distribution of an increased number of minima with lower energy barriers indicated structural changes that could increase the mobility and conformational lability of molecules.

Third, most of the methionine mutations have destabilized the structure of inteins, with the exception of A165M. Any replacement in the core structure of inteins could significantly change the structure and completely block splicing in the case of I151 M and T198A. The dual effect of the extended structure of methionine molecule was revealed in the case of double mutations A162M + A165M with destabilization of the intein C core structure, and in the case of single A165M mutation, methionine has stabilized the C-part catalytic center. A similar effect of histidine extension structure was obtained in the case of A168H.

These new insights into the modification of intein Ssp DnaE could be further used for the structural analysis of intein N and C core interactions. Additional experimental evidence for the modification of the intein C-part to stabilize the catalytic center could be used to obtain new optimized intein pairs. The combination of computational methods for the analysis of the structure and dynamics of intein interactions would significantly accelerate the development of improved versions of intein-mediated trans-splicing systems.

This methodology could be used in many biotechnology applications, such as protein assembly and purification, as a basis for visualizing protein–protein interactions, and as a technology for advanced gene therapy. For instance, in our next research, we will use the obtained results as improved solutions to overcome the limitations of AAV packaging with optimized trans-splicing efficiency. These findings could assist in the discovery of new approaches to create more efficient transgene delivery systems and genome editing systems.

## Supplementary Material



## Notes

Oligonucleotide mutagenesis primers for the C-part of Ssp DnaE (Table S1); free energy surfaces after molecular metadynamic simulations with plot (Figure S1–S6) values of energy minima (Table S2–S7), and structural changes (Figure S7–S11) (PDF)

## Abbreviations

GFP: green fluorescent protein
HEK293: human embryonic kidney 293
DNA: DNA
PCR: polymerase chain reaction
AAV: adeno-associated viruses
cDNA: coding DNA
DMD: Duchenne muscular dystrophy
DYSF: dysferlin
USH2A: usher syndrome type II A
ABCA: ATP-binding cassette 4
EYS: eyes shut homologue
F8/VIII: factor VIII of blood coagulation
ABE: adenine base editor
CBE: cytosine base editor
NMR: nuclear magnetic resonance
SASA: solvent-accessible surface area method
VSGB: variable surface generalized Born model
OPLS4: optimized potential for the liquid simulation model
LBFGS: limited Broyden–Fletcher–Goldfarb–Shanno algorithm
NVT: canonical ensemble amount of substance (N), volume (V), temperature (T)
NPT: canonical ensemble amount of substance (N), pressure (P), temperature (T)
RMSD: root-mean squared deviation
FACS: fluorescence-activated cell sorting
FSC: forward scatter
SSC: side scatter

## References

[1] Wang H., Wang L., Zhong B., Dai Z. (2022). Protein Splicing of
Inteins: A Powerful Tool in Synthetic Biology. Front. Bioeng. Biotechnol..

[2] Shah, N. H. ; Stevens, A. J. Identification, Characterization, and Optimization of Split Inteins. In Methods in Molecular Biology; Springer Nature, 2020; Vol. 2133, pp 31–54. 32144662 10.1007/978-1-0716-0434-2_3

[3] Topilina N. I., Mills K. V. (2014). Recent Advances in in Vivo Applications
of Intein-Mediated
Protein Splicing. Mobile DNA.

[4] Shi C., Tarimala A., Meng Q., Wood D. W. (2014). A General Purification
Platform for Toxic Proteins Based on Intein Trans-Splicing. Appl. Microbiol. Biotechnol..

[5] Tornabene P., Trapani I. (2020). Can Adeno-Associated
Viral Vectors Deliver Effectively
Large Genes?. Human Gene Ther..

[6] Ray D. M., Flood J. R., David Y. (2023). Harnessing
Split-Inteins as a Tool
for the Selective Modification of Surface Receptors in Live Cells. Chembiochem.

[7] Zong H., Han L., Chen J., Pan Z., Wang L., Sun R., Ding K., Xie Y., Jiang H., Lu H., Gilly J., Zhang B., Zhu J. (2022). Kinetics Study of the
Natural Split Npu DnaE Intein in the Generation of Bispecific IgG
Antibodies. Appl. Microbiol. Biotechnol..

[8] Tornabene P., Trapani I., Minopoli R., Centrulo M., Lupo M., de Simone S., Tiberi P., Dell’Aquila F., Marrocco E., Iodice C., Iuliano A., Gesualdo C., Rossi S., Giaquinto L., Albert S., Hoyng C. B., Polishchuk E., Cremers F. P. M., Surace E. M., Simonelli F. (2019). Intein-Mediated Protein Trans-Splicing Expands Adeno-Associated Virus
Transfer Capacity in the Retina. Sci. Transl.
Med..

[9] Kolesnik V. V., Nurtdinov R. F., Oloruntimehin E. S., Karabelsky A. V., Malogolovkin A. S. (2024). Optimization Strategies and Advances in the Research
and Development of AAV-Based Gene Therapy to Deliver Large Transgenes. Clin. Transl. Med..

[10] Truong D.-J. J., Kühner K., Kühn R., Werfel S., Engelhardt S., Wurst W., Ortiz O. (2015). Development
of an Intein-Mediated Split–Cas9 System for Gene Therapy. Nucleic Acids Res..

[11] Huang T. P., Newby G. A., Liu D. R. (2021). Precision
Genome Editing Using Cytosine
and Adenine Base Editors in Mammalian Cells. Nat. Protoc..

[12] Zeng H., Yuan Q., Peng F., Ma D., Lingineni A., Chee K., Gilberd P., Osikpa E. C., Sun Z., Gao X. (2023). A Split and Inducible Adenine Base Editor for Precise
in Vivo Base
Editing. Nat. Commun..

[13] Peters C. W., Hanlon K. S., Ivanchenko M. V., Zinn E., Linarte E. F., Li Y., Levy J. M., Liu D. R., Kleinstiver B. P., Indzhykulian A. A., Corey D. P. (2023). Rescue of Hearing by Adenine Base
Editing in a Humanized Mouse Model of Usher Syndrome Type 1F. Mol. Ther..

[14] Wu S. (2024). Base Editing Effectively
Prevents Early-Onset Severe Cardiomyopathy
in Mybpc3Mutant Mice. Cell Res..

[15] Villiger L., Grisch-Chan H. M., Lindsay H., Ringnalda F., Pogliano C. B., Allegri G., Fingerhut R., Häberle J., Matos J., Robinson M. D., Thöny B., Schwank G. (2018). Treatment of a Metabolic Liver Disease by in Vivo Genome
Base Editing in Adult Mice. Nat. Med..

[16] Villiger L., Rothgangl T., Witzigmann D., Oka R., Lin P. J. C., Qi W., Janjuha S., Berk C., Ringnalda F., Beattie M. B., Stoffel M., Thöny B., Hall J., Rehrauer H., van Boxtel R., Tam Y. K., Schwank G. (2021). In Vivo Cytidine Base Editing of
Hepatocytes without Detectable Off-Target Mutations in RNA and DNA. Nature Biomed. Eng..

[17] Liu Q., Chen Y., Hu S., Liu W., Xie D., Yang X., Huang W., Liu S., Chen X., Liu H., Huang J. (2023). Screening an Effective
Dual-Adeno-Associated Virus
Split-Cytosine Base Editor System for C-To-T Conversion in Vivo. Human Gene Ther..

[18] Yun, S. Engineered CRISPR-Base Eds. as a Permanent Treatment for Familial Dysautonomia. In BioRxiv; Preprint, 2024.

[19] Zhi S., Chen Y., Wu G., Wen J., Wu J., Liu Q., Li Y., Kang R., Hu S., Wang J., Liang P., Huang J. (2022). Dual-AAV Delivering
Split Prime Editor
System for in Vivo Genome Editing. Mol. Ther..

[20] She K., Liu Y., Zhao Q. (2023). Dual-AAV Split Prime Editor Corrects the Mutation
and Phenotype in Mice with Inherited Retinal Degeneration. Signal Transduction Targeted Ther..

[21] Wei R., Yu Z., Ding L., Lu Z., Yao K., Zhang H., Huang B., He M., Ma L. (2025). Improved Split Prime
Editors Enable Efficient in Vivo Genome Editing. Cell Rep..

[22] Xia H.-F., Luo J.-P., Yu S.-R., Zhou T.-J. (2022). Modification
of
C-Segment of Cfa DnaE Split Intein for Improving Clean-In-Place in
Chromatography Process. Biotechnology Prog..

[23] Humberg, C. A Cysteine-Less and Ultra-Fast Split Intein Rationally Engineered from Being Aggregation-Prone to Highly Efficient in Protein Trans-Splicing. In BioRxiv; Preprint, 2025. 10.1038/s41467-025-57596-x PMC11923092 40108172

[24] Hiltunen M.
K., Beyer H. M., Iwaï H. (2021). Mini-Intein Structures from Extremophiles
Suggest a Strategy for Finding Novel Robust Inteins. Microorganisms.

[25] Boral S., Maiti S., Basak A. J., Lee W., De S. (2020). Structural,
Dynamic, and Functional Characterization of a DnaX Mini-Intein Derived
from Spirulina Platensis Provides Important Insights into Intein-Mediated
Catalysis of Protein Splicing. Biochemistry.

[26] Heikkinen H. A., Aranko A. S., Iwaï H. (2022). The NMR Structure of the Engineered
Halophilic DnaE Intein for Segmental Isotopic Labeling Using Conditional
Protein Splicing. J. Magn. Reson..

[27] Shah N. H., Eryilmaz E., Cowburn D., Muir T. W. (2013). Naturally Split
Inteins Assemble through a “Capture and Collapse” Mechanism. J. Am. Chem. Soc..

[28] Ludwig C., Schwarzer D., Mootz H. D. (2008). Interaction Studies and Alanine Scanning
Analysis of a Semi-Synthetic Split Intein Reveal Thiazoline Ring Formation
from an Intermediate of the Protein Splicing Reaction. J. Biol. Chem..

[29] Wasmuth A., Ludwig C., Mootz H. D. (2013). Structure-Activity Studies on the
Upstream Splice Junction of a Semisynthetic Intein. Bioorg. Med. Chem..

[30] Stevens A. J., Brown Z. Z., Shah N. H., Sekar G., Cowburn D., Muir T. W. (2016). Design of a Split Intein with Exceptional
Protein Splicing
Activity. J. Am. Chem. Soc..

[31] Eryilmaz E., Shah N. H., Muir T. W., Cowburn D. (2014). Structural and Dynamical
Features of Inteins and Implications on Protein Splicing. J. Biol. Chem..

[32] Pietrokovski S. (2001). Intein Spread
and Extinction in Evolution. Trends Genet..

[33] Shah N. H., Dann G. P., Vila-Perelló M., Liu Z., Muir T. W. (2012). Ultrafast
Protein Splicing Is Common among Cyanobacterial Split Inteins: Implications
for Protein Engineering. J. Am. Chem. Soc..

[34] RCSB PDB: Homepage. https://www.rcsb.org/ (accessed June 01, 2024).

[35] Sastry G. M., Adzhigirey M., Day T., Annabhimoju R., Sherman W. (2013). Protein and Ligand Preparation: Parameters,
Protocols,
and Influence on Virtual Screening Enrichments. J. Comput. Mol. Des..

[36] Jacobson M. P., Friesner R. A., Xiang Z., Honig B. (2002). On the Role of the
Crystal Environment in Determining Protein Side-Chain Conformations. J. Mol. Biol..

[37] Lu C., Wu C., Ghoreishi D., Chen W., Wang L., Damm W., Ross G. A., Dahlgren M. K., Russell E., Von Bargen C. D., Abel R., Friesner R. A., Harder E. D. (2021). OPLS4:
Improving
Force Field Accuracy on Challenging Regimes of Chemical Space. J. Chem. Theory Comput..

[38] Ramachandran G. N., Ramakrishnan C., Sasisekharan V. (1963). Stereochemistry
of Polypeptide Chain
Configurations. J. Mol. Biol..

[39] Kozakov D., Brenke R., Comeau S. R., Vajda S. (2006). PIPER: An FFT-Based
Protein Docking Program with Pairwise Potentials. Proteins.

[40] Brovin A., Minskaia E., Sabantsev M., Chuvpilo S., Karabelsky A. (2024). Protein Trans-Splicing:
Optimization of Intein-Mediated GFP Assembly as a Model for the Development
of Gene Therapy. Front. Bioeng. Biotechnol..

[41] Perler F. B. (2002). InBase:
The Intein Database. Nucleic Acids Res..

[42] Dearden A. K., Nayak S. K. (2015). Proteins: Ssp DnaE Intein. Multiscale
Model. Biomech. Mechanobiol..

[43] Nichols N. M., Evans T. C. (2004). Mutational Analysis of Protein Splicing, Cleavage,
and Self-Association Reactions Mediated by the Naturally Split Ssp
DnaE Intein. Biochemistry.

[44] Kick L. M., Harteis S., Koch M. F., Schneider S. (2017). Mechanistic
Insights into Cyclic Peptide Generation by DnaE Split-Inteins through
Quantitative and Structural Investigation. ChemBioChem.

[45] Panda S., Rout M., Mishra S., Turuk J., Pati S., Dehury B. (2023). Molecular Docking and MD Simulations Reveal Protease
Inhibitors Block the Catalytic Residues in Prp8 Intein of Aspergillus
Fumigatus: a Potential Target for Antimycotics. J. Biomol. Struct. Dyn..

[46] Cronin M., Coolbaugh M. J., Nellis D., Zhu J., Wood D. W., Nussinov R., Ma B. (2015). Dynamics Differentiate between Active
and Inactive Inteins. European J. Med. Chem..

[47] Karp J. M., Eryilmaz E., Cowburn D. (2015). Correlation
of Chemical Shifts Predicted
by Molecular Dynamics Simulations for Partially Disordered Proteins. J. Biomol. NMR.

[48] Wetie A. G. N., Sokolowska I., Woods A. G., Roy U., Deinhardt K., Darie C. C. (2014). Protein–Protein Interactions:
Switch from Classical
Methods to Proteomics and Bioinformatics-Based Approaches. Cell. Mol. Life Sci..

[49] Sekar G., Stevens A. J., Mostafavi A. Z., Sashi P., Muir T. W., Cowburn D. (2022). A Conserved Histidine
Residue Drives Extein Dependence
in an Enhanced Atypically Split Intein. J. Am.
Chem. Soc..

[50] Li R., Jing Q., She K., Wang Q., Jin X., Zhao Q., Su J., Song L., Fu J., Wu X., Xu Q., Lu F., Wei Y., Yang Y. (2023). Split AAV8Mediated
ABCA4 Expression for Gene Therapy of Mouse Stargardt Disease (STGD1). Human Gene Ther..

[51] Purkayastha A., Kang T. J. (2019). Stabilization of
Proteins by Covalent Cyclization. Biotechnol.
Bioprocess. Eng..

[52] Oeemig J. S., Zhou D., Kajander T., Wlodawer A., Iwaï H. (2012). NMR and Crystal
Structures of the Pyrococcus Horikoshii RadA Intein Guide a Strategy
for Engineering a Highly Efficient and Promiscuous Intein. J. Mol. Biol..

[53] Oeemig J. S., Beyer H. M., Aranko A. S., Mutanen J., Iwaï H. (2020). Substrate
Specificities of Inteins Investigated by QuickDrop-Cassette Mutagenesis. FEBS Lett..

[54] Carvajal-Vallejos P., Pallissé R., Mootz H. D., Schmidt S. R. (2012). Unprecedented Rates
and Efficiencies Revealed for New Natural Split Inteins from Metagenomic
Sources. J. Biol. Chem..

[55] Beyer H. M., Mikula K. M., Li M., Wlodawer A., Iwaï H. (2020). The Crystal
Structure of the Naturally Split Gp41–1 Intein Guides the Engineering
of Orthogonal Split Inteins Fromcis-Splicing Inteins. FEBS J.

[56] Mariano A., Di Cristofano S., Raimondo D., D’Abusco A. S. (2024). Split Gp41–1
Intein Splicing as a Model to Evaluate the Cellular Location of the
Oncosuppressor Maspin in an in Vitro Model of Osteosarcoma. Cell Biochem. Funct..

[57] Kothawala A., Kilpatrick K., Novoa J. A., Segatori L. (2012). Quantitative Analysis
of α-Synuclein Solubility in Living Cells Using Split GFP Complementation. PLoS One.

