# Supplementary material for: Application of Rational Design and Molecular Metadynamics for the Estimation of Changes in Trans-Splicing Efficiency during the Mutagenesis of Ssp DnaE Intein
Source: ACS Bio Med Chem Au. 2025 Aug 8;5(4):738–52. doi: 10.1021/acsbiomedchemau.5c00091 (PMC12371501; doi:10.1021/acsbiomedchemau.5c00091)
Supplement: Supplementary file 1 [file bg5c00091_si_001.pdf]

Supporting Information

Application of rational design and molecular metadynamics for estimation of changes in trans-splicing efficiency during mutagenesis of SspDnaE intein

Matvei O. Sabantsev[1], Andrew N. Brovin*[1], Maxim A. Gureev[2], Yuri B. Porozov[2], Sergey A. Chuvpilo[1], Alexander V. Karabelsky[1]

1. Gene Therapy Department, Research Center for Translational Medicine, Sirius University of Science and Technology, Sirius, 354349, Russia

2. Laboratory of bio-and chemoinformatics, HSE University, Soyuza Pechatnikov str.16, Saint-Petersburg, Russia, 190121

* Correspondence to Andrew N. Brovin - Gene Therapy Department, Research Center for Translational Medicine, Sirius University of Science and Technology, Sirius, 354349, Russia,

orcid: https://orcid.org/0009-0001-7517-4924 E-mail: brovin.an@talantiuspeh.ru

Table S1. Oligonucleotide mutagenesis primers for C part of Ssp Dna-E.

Primer name	Primer sequence
BamHI_Ssp_C_1F	AATTTAGGATCCATGGTTAAGGTGATTGGAAGACGTTCT
Ssp_C_eGFP70_F	CTAATGGTGCCATCGCTGCCAATTGCTTCAGCCGCTACCCCGACCACATG
SSPC_I151M_R	ATTGGCAGCGATGGCACCATTAGCGAGAAGAAAGTTGTGGTCTTGTGGCAAT CCCATATCGAAGATCCTTTGAACACCAAGAGAACGTCTTCCAATCACCTT
SSPC_F159M_R	ATTGGCAGCGATGGCACCATTAGCGAGAAGCATGTTGTGGTCTTGTGGCAAT CCGATATCGAAGATCCTTTGAACACCAAGAGAACGTCTTCCAATCACCTT
SSPC_A162M_R	ATTGGCAGCGATGGCACCATTCATGAGAAGAAAGTTGTGGTCTTGTGGCAAT CCGATATCGAAGATCCTTTGAACACCAAGAGAACGTCTTCCAATCACCTT
SSPC_A165M_R	ATTGGCAGCGATCATACCATTAGCGAGAAGAAAGTTGTGGTCTTGTGGCAAT CCGATATCGAAGATCCTTTGAACACCAAGAGAACGTCTTCCAATCACCTT
SSPC_ A162M+ A165M _R	ATTGGCAGCGATCATACCATTCATGAGAAGAAAGTTGTGGTCTTGTGGCAAT CCGATATCGAAGATCCTTTGAACACCAAGAGAACGTCTTCCAATCACCTT
SSPC_ A162F+ A165M _R	ATTGGCAGCGATCATACCATTGAAGAGAAGAAAGTTGTGGTCTTGTGGCAAT CCGATATCGAAGATCCTTTGAACACCAAGAGAACGTCTTCCAATCACCTT
eGFP_70_F	TGCTTCAGCCGCTACCCCG
eGFP_HindIII_R	AATTATAAGCTTTTACTTGTACAGCTCGTCCATGCCG

Table S2. Gibbs free energy (ΔG) parameters of conformational states of the Ssp DnaE intein in its original state without mutations.

Native intein Ssp Dna E with fused GFP					
Energy minima	ΔG (kcal/mol)	CV1	CV2	barrier ΔΔG (kcal/mol)	
1	-30,75	15,85	6,09	1→2	+18,75
2	-15,91	18,51	8,28	2→3	+4,91
3	-17,37	18,14	10,99	3→4	+10,37
4	-13,73	19,03	14,96		

Table S3. Gibbs free energy (ΔG) parameters of conformational states of the dnaE intein with a single mutation A165M.

A165M intein with fused GFP					
Energy minima	ΔG (kcal/mol)	CV1	CV2	barrier ΔΔG (kcal/mol)	
1	-18.04	13.98	6.47	1→2	+4.24
2	-18.62	15.33	7.43	2→3	+4.72
3	-17.75	17.25	8.82	3→4	+10.93
4	-12.83	20.84	13.16	4→5	+6.03
5	-10.10	19.87	18.55	4→6	+9.63
6	-5.74	29.60	14.22	4→7	+8.33
7	-4.97	24.46	16.16		

Table S4. Gibbs free energy (ΔG) parameters of conformational states of the dnaE intein with a double mutation A162M+A165M.

A162M+A165M intein with fused GFP					
Energy minima	ΔG (kcal/mol)	CV1	CV2	barrier ΔΔG (kcal/mol)	
1	-22,56	13,36	6,32	1→2	+10.96
2	-14,76	17,40	6,98	2→3	+3.76
3	-15,57	17,41	8,78	3→4	+3.23
4	-16,62	14,94	9,14	4→5	+4.82
5	-15,31	18,83	11,01	5→6	+11.08
6	-8,67	29,93	14,80		

Table S5. Gibbs free energy (ΔG) parameters of conformational states of the dnaE intein with a single mutations I151M.

I151M intein with fused GFP					
Energy minima	ΔG (kcal/mol)	CV1	CV2	barrier ΔΔG (kcal/mol)	
1	-21,12	15,24	6,21	1→2	+6.02
2	-24,30	14,48	7,81	2→3	+9.20
3	-22,26	17,33	6,37	1→3	+2.03
4	-21,33	16,75	10,11	2→4	+5.03
5	-14,02	20,41	8,01	4→5	+8.73

Table S6 Gibbs free energy (ΔG) parameters of conformational states of the dnaE integrated under mutations A168H.

A168H intein with fused GFP					
Energy minima	ΔG (kcal/mol)	CV1	CV2	barrier ΔΔG (kcal/mol)	
1	-20.73	13.97	6.13	1→2	+4.73
2	-16.53	15.84	6.42	2→3	+6.52
3	-14.32	14.52	9.11	3→4	+6.32
4	-15.14	17.85	8.66	4→5	+0.52
5	-14.22	20.12	9.31	5→6	+0.64
6	-15.76	18.50	10.39	6→7	+7.74
7	-8.16	17.77	13.63	7→8	+4.04
8	-9.00	21.17	15.12	8→9	+4.26
9	-9.26	22.70	13.79		

Table S7 Gibbs free energy (ΔG) parameters of conformational states of the dnaE integrated under mutations T198A.

T198A intein with fused GFP					
Energy minima	ΔG (kcal/mol)	CV1	CV2	barrier ΔΔG (kcal/mol)	
1	-31.86	16.80	6.23	1→2	+19,86
2	-15.97	18.45	9.38	1→3	+19,32
3	-13.28	22.77	8.63		

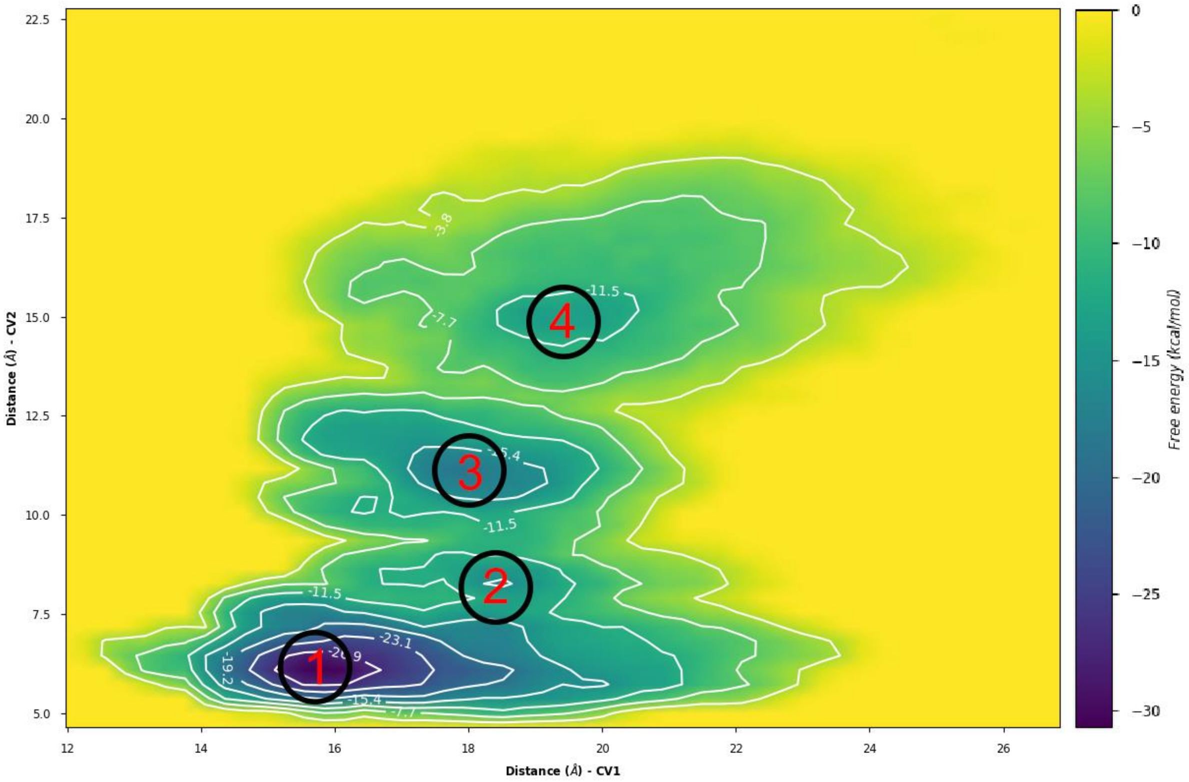

Figure S1. Free energy surface of Ssp DnaE intein in its original state without mutations, 1-4 – energy minima that was described in Table S2

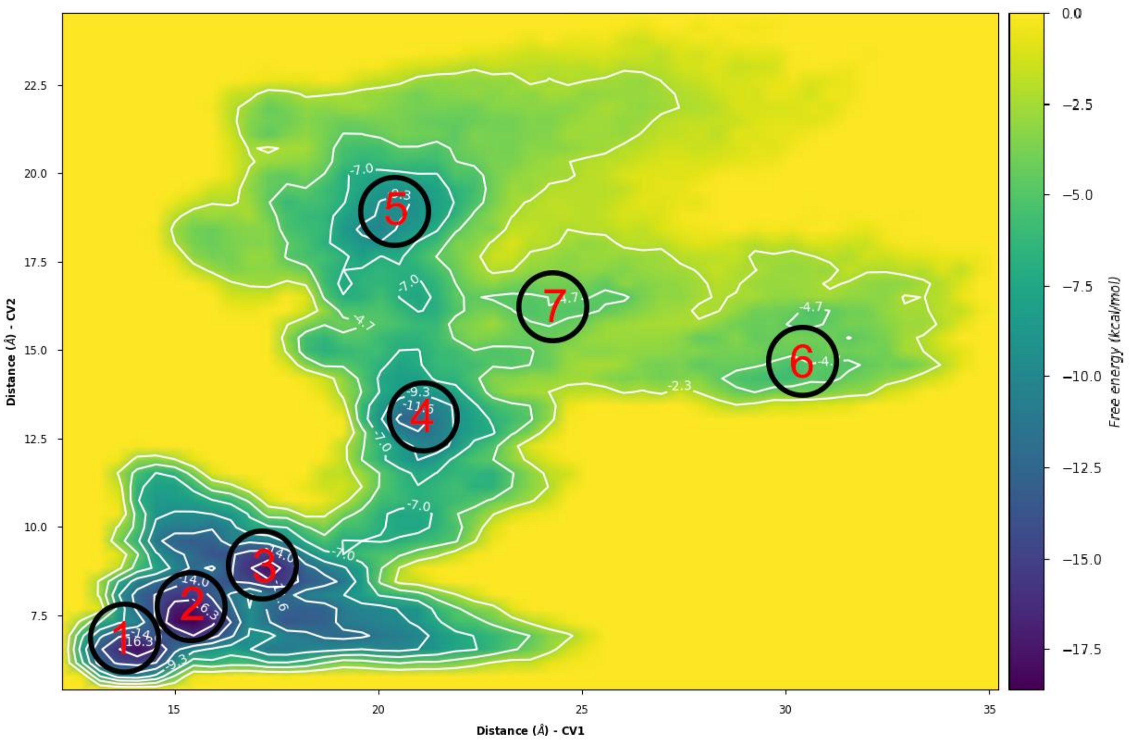

Figure S2. Free energy surface of the Ssp DnaE intein with A165M mutation, 1-7 – energy minima that was described in Table S3

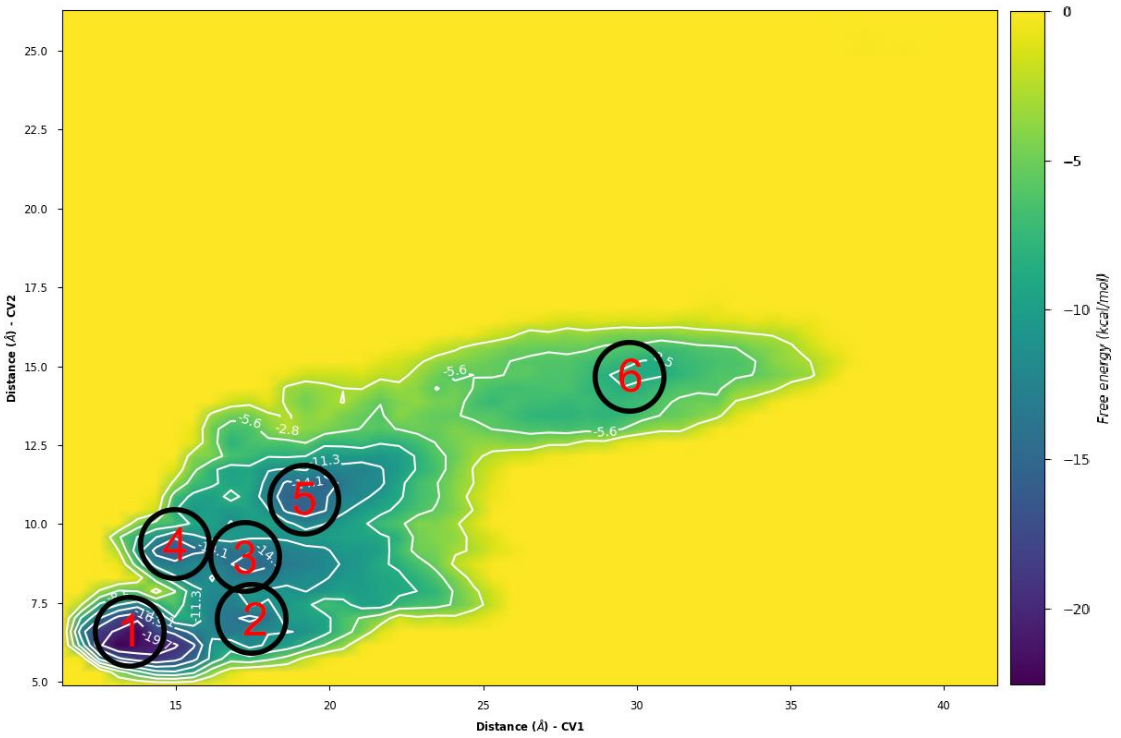

Figure S3. Free energy surface of the Ssp DnaE intein with double A162M+A165M mutations, 1-6 – energy minima that was described in Table S4

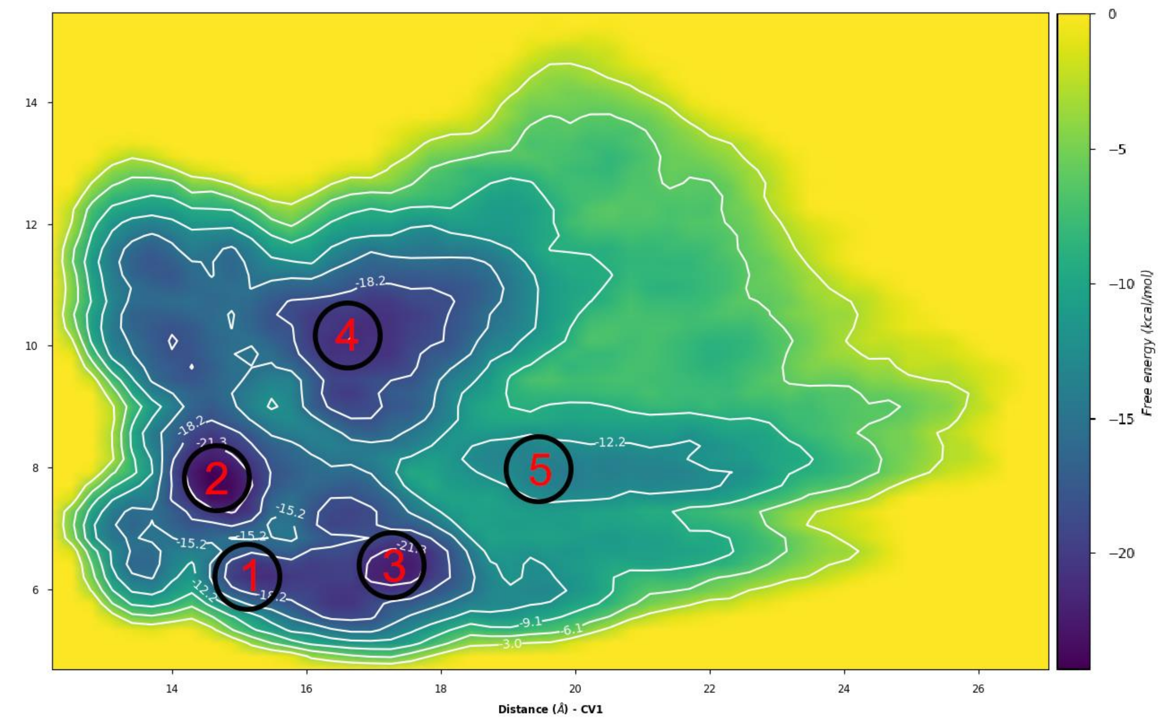

Figure S4. Free energy surface of the Ssp DnaE intein with I151M mutation, 1-5 – energy minima that was described in Table S5

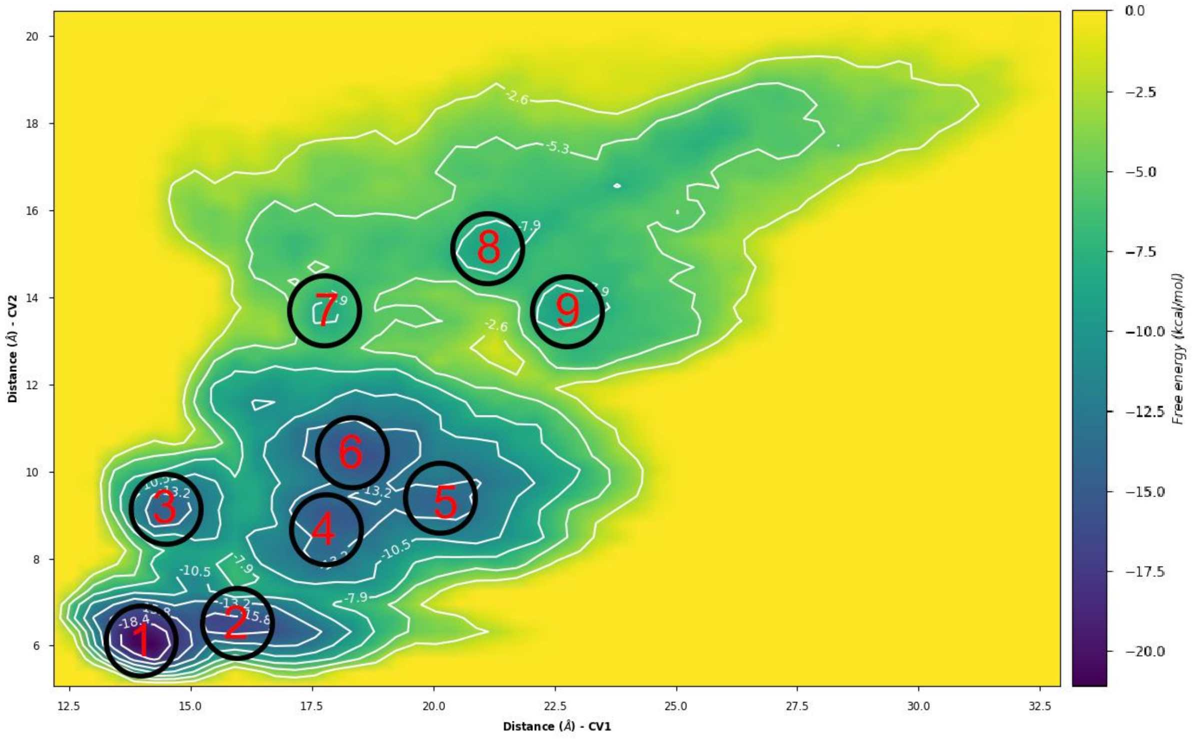

Figure S5. Free energy surface of the Ssp DnaE intein with A168H mutation, 1-9 – energy minima that was described in Table S6

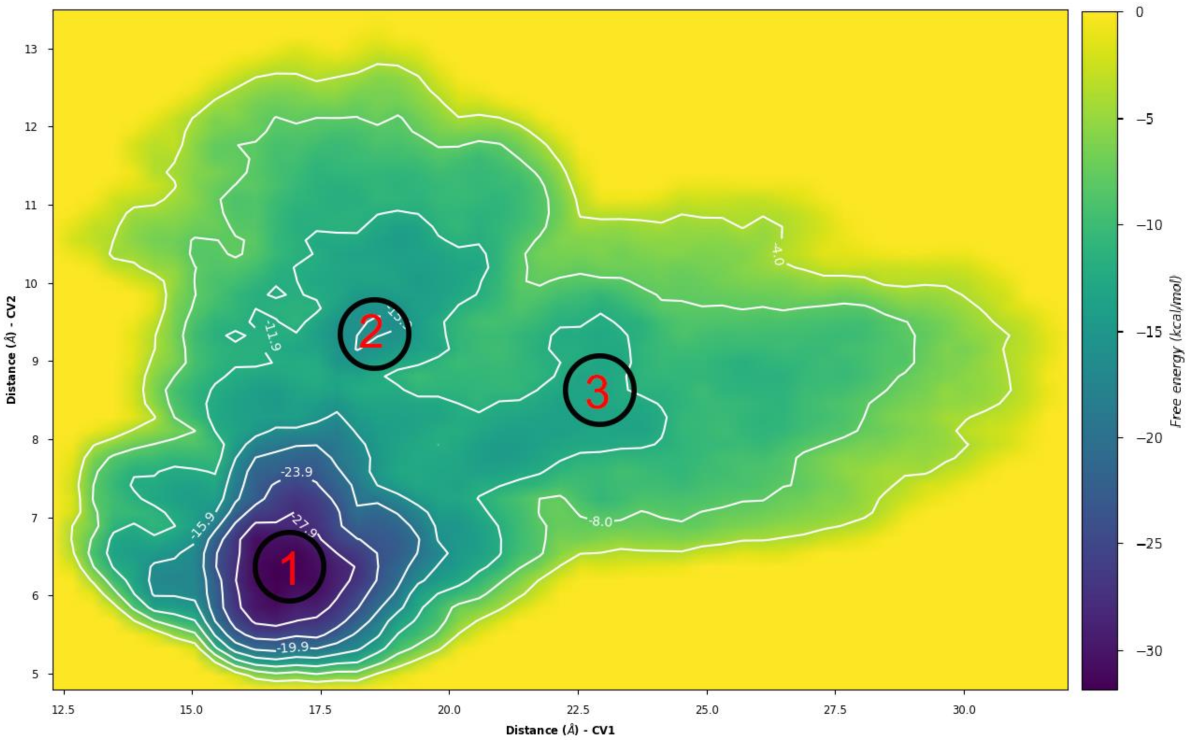

Figure S6. Free energy surface of the Ssp DnaE intein with T198A mutation, 1-3 – energy minima that was described in Table S7

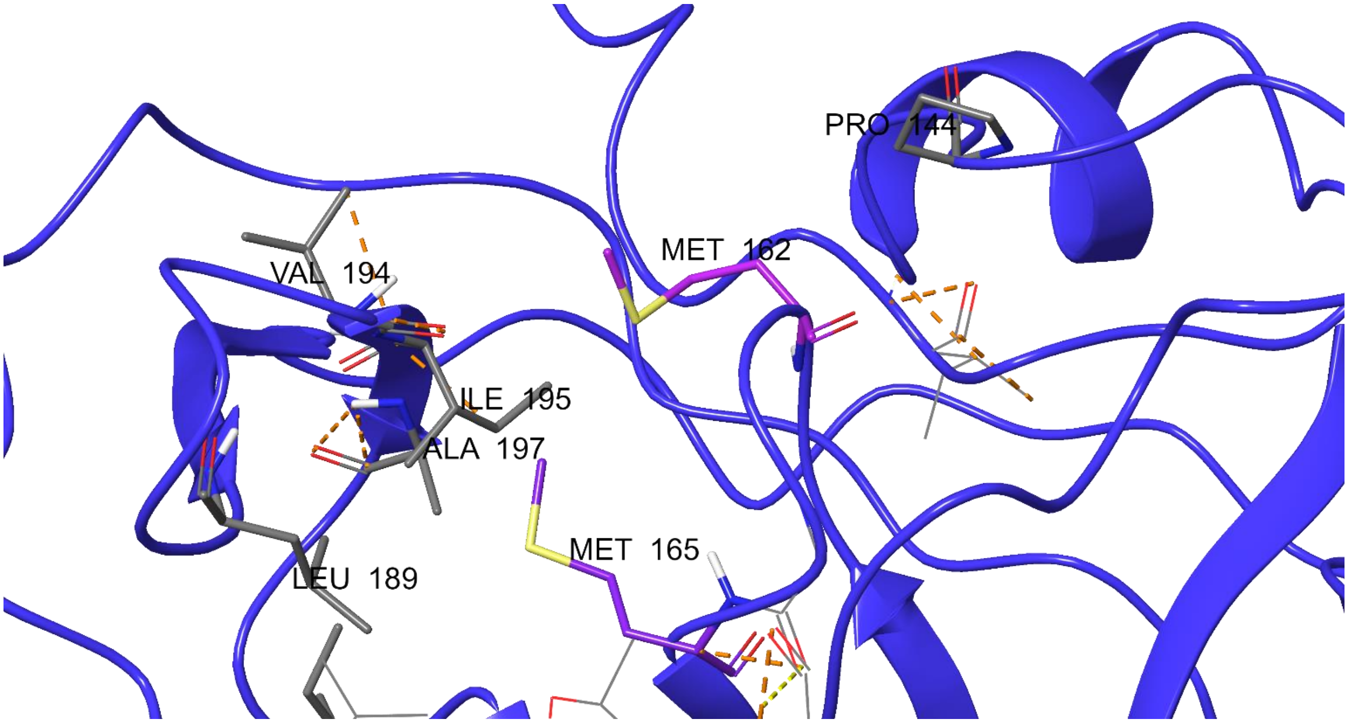

Figure S7. Destabilisation of the Ssp DnaE C-part with double A162M+A165M mutations

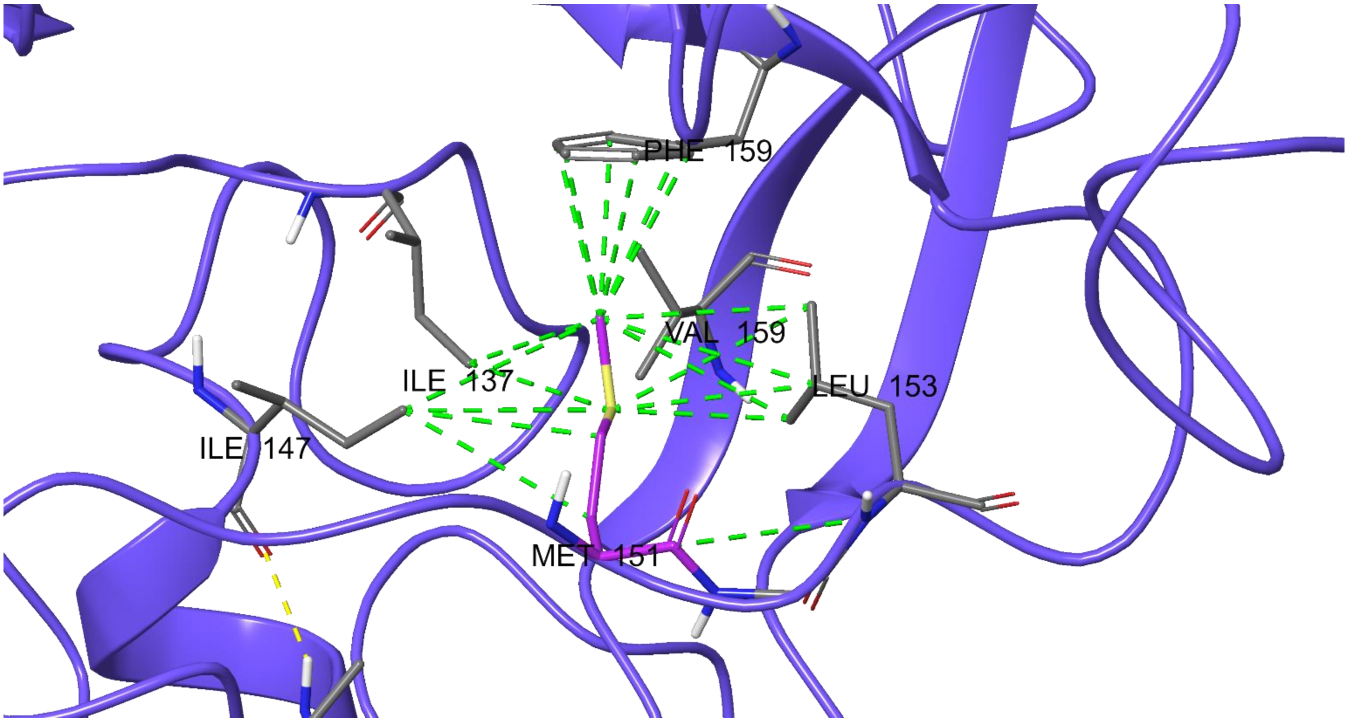

Figure S8. Over-stabilised Ssp DnaE C-part molecule with increased number of lipophilic contacts in structure with mutation I151M; dashed lines: green – lipophilic contacts, yellow – H-bonds, magenta – salt bridge

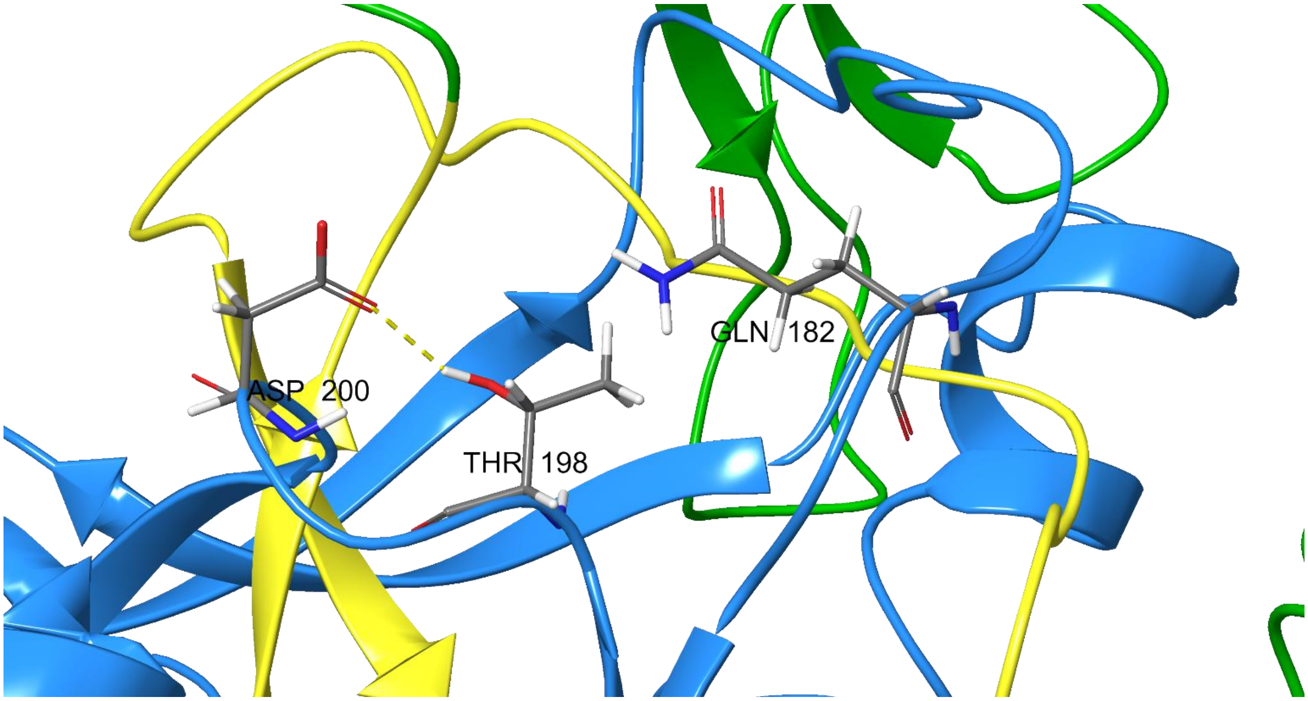

Figure S9. Interactions between amino acids in the structure of the intein at position of T198 residue before mutation. Threonine (T198) acting as a bridge realising transient contacts between aspartic acid (D200) and glutamine (Q182). Dashed lines: yellow – hydrogen bond.

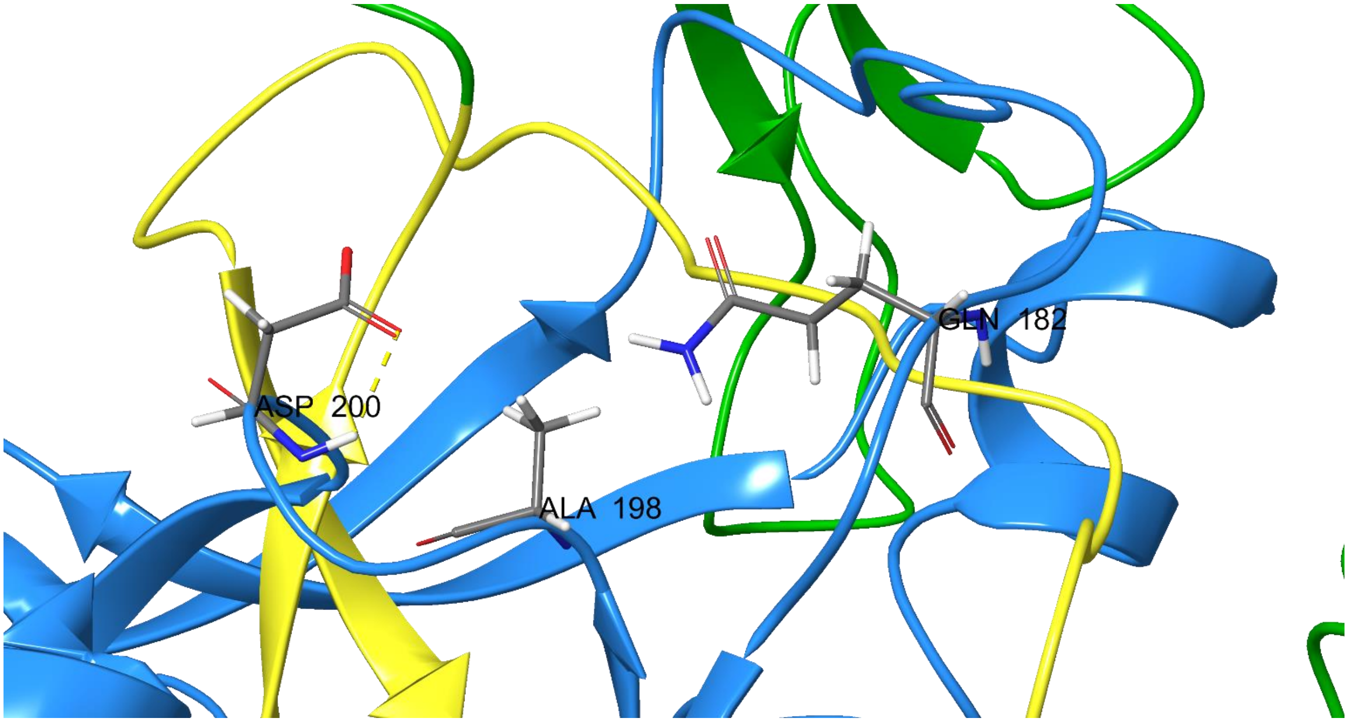

Figure S10. Interactions between amino acids in the structure of the intein with mutation T198A. Threonine bridge was disrupted with alanine replacement. Dashed lines: yellow – hydrogen bond.

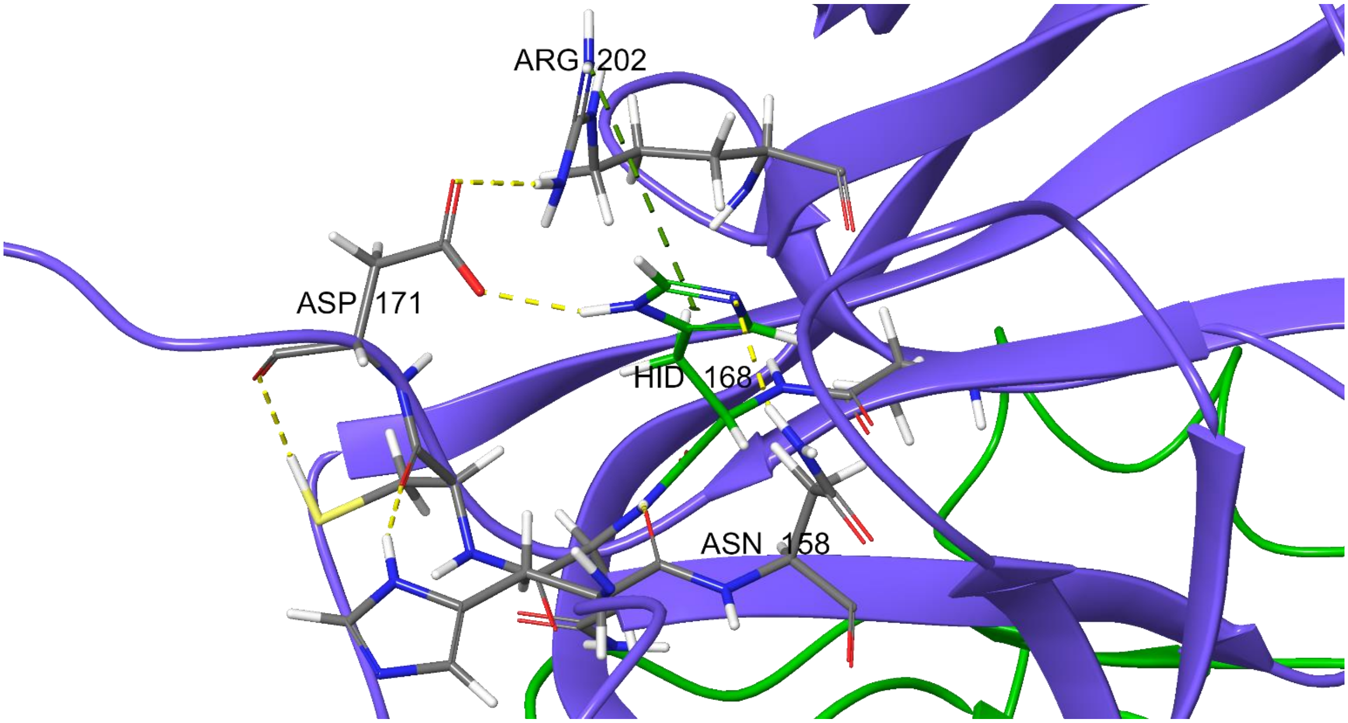

Figure S11. Stabilised Ssp DnaE C-part molecule with A168H mutation that has formed π-cation contacts with A:R202 and hydrogen bonds with B:D171 and B:N 158. Dashed lines: yellow – hydrogen bond, green – π-cation interaction.
